# Evidence‐based safety profile of oral ketorolac in adults: Systematic review and meta‐analysis

**DOI:** 10.1002/prp2.70033

**Published:** 2024-11-23

**Authors:** Vicente Esparza‐Villalpando, Gladys Ortiz‐Barroso, David Masuoka‐Ito

**Affiliations:** ^1^ Health Sciences Center, Stomatology Department Autonomous University of Aguascalientes Aguascalientes Mexico; ^2^ Health Sciences Center Autonomous University of Aguascalientes Aguascalientes Mexico

**Keywords:** meta‐analysis, oral ketorolac, risk ratio, safety profile

## Abstract

The primary objective of the present review was to report the safety profile of oral ketorolac in adults using the systematic review and meta‐analysis methodology based on clinical trials. The present study is a PRISMA‐based systematic review and risk ratio (RR) meta‐analysis of the adverse events reported in clinical trials that used oral ketorolac; the review includes 50 clinical trials. The RR for the comparison of a single intake of oral ketorolac versus placebo, including all types of adverse events, was RR = 2.59, IC95% (1.5102; 4.4360) with *p* = 0.02, the RR for the comparison of a multiple intakes of oral ketorolac versus placebo for all types of adverse events was RR = 1.39, IC95% (0.95; 2.05) with *p* = 0.093, the RR for the comparison of a single intake of oral ketorolac versus active drugs for all types of adverse events was RR = 0.61, IC95% (0.49; 0.77) with *p* < 0.0001, the RR for the comparison of multiple intakes of oral ketorolac versus active drugs for all types of adverse events was RR = 0.78, IC95%(0.65; 0.93) with *p* = 0.006. Multiple intakes of 5, 10, or 20 mg of oral ketorolac, in treatment over 1–10 days, do not increase the risk of adverse events compared to placebo and show a tendency to reduce the risk of adverse events compared to active drugs. When a single intake of ketorolac (5, 10, 20, or 30 mg) is compared to a placebo, the risk increases only for trivial and mild adverse events.

## 1 INTRODUCTION

Postoperative pain management involves multiple modes of analgesia, including NSAIDs, opioids, multimodal analgesia, and patient control analgesia (PCA). These can also be used with adjuncts like TENS, coadjuvant drugs, and cold therapy.^1^ Therefore, the analgesic selection, the administration route, and its potential adverse events on widely available drugs remain a challenge to clinicians seeking pain control.^1^, ^2^

Ketorolac is a useful NSAIDS for pain control. This drug is a thrometamine salt that can be administrated in different routes, including intramuscular, intravenous, sublingual, and oral.^3^ The absorption of oral ketorolac is rapid, its bioavailability is approximately 80%–100% after administration, its action duration is about 6–8 h, and it has a 1‐week washout period.^3^ Its mechanism of action has been described as the inhibition of COX‐1 (cyclooxygenase) and COX‐2, which decreases the concentration of prostaglandins at inflammation sites.^4^ Therefore, it is an important drug for pain management in various surgical and nonsurgical procedures and can reduce the use of opioids.^4^

The ketorolac analgesic efficacy has been assessed in different clinical contexts^5^, ^6^, ^7^, ^8^ A good response regarding pain control, relatively good tolerance, and safety have been reported. However, the specific safety profile of oral ketorolac remains unclear because most of the studies are focused on other routes of administration.^5^, ^9^

Reports of the adverse events of ketorolac include upper gastrointestinal complications,^10^ renal and cardiovascular function, and bleeding.^11^ Other studies assessing the safety profile of ketorolac in pediatric populations.^6^, ^12^, ^13^ These studies showed differences in the methods used to measure adverse events. Most of them were observational studies with different administration routes, times, and doses and the absence of control of confounders.^10^ Therefore, the safety profile of oral ketorolac remains unclear. The primary objective of the present review was to report the safety profile of oral ketorolac in adults using systematic review and meta‐analysis methodology based on available clinical trial evidence.

## 2 METHODS AND MATERIALS

### 2.1 Methodological design

This study is a systematic review and meta‐analysis and was prepared following the Preferred Reporting Items for Systematic Reviews and Meta‐analysis (PRISMA) principles used by Liberati et al.,^14^ the Cochrane Group fundamentals, and the recommendations made by Higgins and Green.^15^

### 2.2 Selection criteria

Articles reporting adverse events related to the oral administration of ketorolac under postoperative pain conditions in multiple clinical scenarios in the adult population were considered eligible (including randomized clinical trials [RCTs], parallel groups, and crossover or split‐mouth designs). Narrative literature reviews, observational studies, case reports, in vitro or animal studies, abstracts, and incomplete and unpublished reports were excluded. Studies with ketorolac groups combined with other drugs were also excluded. Intervention, control, and outcome parameters were selected following the Population, Interventions, Control, and Outcome (PICO) question:

#### 2.2.1 Population

Postoperative pain conditions in the adult population.

#### 2.2.2 Interventions

Oral administration of ketorolac.

#### 2.2.3 Control

Placebo/other analgesic treatment.

#### 2.2.4 Outcome

Odds ratio, risk ratio, and presence/absence of adverse events.

### 2.3 Search strategy and data extraction

The literature search of relevant references was made between November 2023 and August 2024 in electronic databases without publication‐date restrictions, including only articles/abstracts in English. PubMed, Cochrane Library, EMBASE (Elsevier Science), Google Scholar, SCOPUS, Web of Science, ScienceDirect, EBSCOhost, Wiley Online Library, and Springer databases were analyzed. The search algorithm was: (“*Ketorolac [MeSH]/oral” AND* “*Ketorolac [MeSH]/safety” AND* “*Ketorolac [MeSH]/adverse effect” AND* “*Ketorolac [MeSH]/lumbar pain” AND* “*Ketorolac [MeSH]/cervical pain” AND* “*Ketorolac [MeSH]/brachialgia” AND* “*Ketorolac [MeSH]/radiculitis” AND* “*Ketorolac [MeSH]/facial paralysis” AND* “*Ketorolac [MeSH]/trigeminal neuralgia” AND* “*Ketorolac [MeSH]/intercostal neuralgia” AND* “*Ketorolac [MeSH]/herpetic neuralgia” AND* “*Ketorolac [MeSH]/alcoholic neuropathy” AND* “*Ketorolac [MeSH]/diabetic neuropathy” AND* “*Ketorolac [MeSH]/carpal tunnel syndrome” AND* “*Ketorolac [MeSH]/neuropathy” AND* “*Ketorolac [MeSH]/gastrointestinal” AND* “*Ketorolac [MeSH]/hematological” AND* “*Ketorolac [MeSH]/renal” AND* “*Ketorolac [MeSH]/hepatic” AND* “*Ketorolac [MeSH]/hypersensitivity” AND* “*Ketorolac [MeSH]/toxicity” AND* “*Ketorolac [MeSH]/vitamin B complex”*). Additionally, the authors hand‐searched international peer‐reviewed pain and surgery journals.

The authors' names, titles, abstracts, keywords, design, and evaluation period of each article preselected according to the inclusion criteria were objectively and independently screened by GOB and subsequently by MID; VEV resolved any discrepancy in the selection criteria. Selected studies were retrieved as full‐text articles to be rescreened in detail by these same reviewers to confirm whether the studies met the inclusion criteria. Data were extracted independently by GOB and VEV from the selected papers and pooled on a data extraction sheet. The article authors were contacted if the reviewers had any data‐related questions or if additional information was needed; studies not found electronically were excluded.

### 2.4 Statistical analysis

A risk ratio (RR) meta‐analysis was performed using the inverse variance method. The heterogeneity was assessed using Q‐statistics with a chi‐squared distribution, a restricted maximum‐likelihood estimator for tau2, the Q‐Profile method for a confidence interval of tau2, and continuity correction of 0.5 in studies with zero cell frequencies. I^2^ was calculated based on Higgins and Thompson.^16^ When the results of a study had significant heterogeneity, random effects were used; otherwise, common effects were applied. The RR was reported with a confidence interval of 95% (IC95%) for each comparison. The analysis methods were based on Cheng et al.,^17^ and the recommendations for reporting effect sizes using the Cochrane Collaboration.^15^ The analysis was performed using R ver. 4.3.2 and meta package.^18^

### 2.5 Quality appraisal

The methodological quality and validity of the included studies were independently assessed by two reviewers (GOB and VEV), previously calibrated (Cohen's kappa = 0.86) using the Grading of Recommendations Assessment, Development and Evaluation (GRADE)^19^ and Oxford Centre for Evidence‐Based Medicine (OCEMB)^20^ criteria, using the table reported by Pozos‐Guillén et al.^21^, ^22^ Both sets of criteria have been considered suitable for reducing potential biases in terms of the evaluation of quality of the RCT. The reviewers scored each scale section based on their judgment and knowledge to determine the respective section's weight regarding each study's results and conclusions, with individual score points for each section and a possible maximum value of 16.

### 2.6 Variable of adverse event of ketorolac

The term “adverse event” was used to refer to an adverse outcome that can be attributed, with some degree of probability, to a drug's action or that occurs afterward but may or may not be attributable to it.^23^ The severity classification of adverse events suggested by Aronson and Ferner was used to group the adverse events of oral ketorolac.^23^ As those authors specified, there is no satisfactory classification; any classification can be challenging to apply and depends on the patient's perception and the clinician's interpretation. However, to integrate all the described adverse events, the following groups were used:

- Trivial: nuisance value only.
- Mild: some interference with patient function, but the patient's normal function not altered.
- Moderate: symptoms marked, but involvement in vital organ systems moderate; symptoms produced some degree of impairment to function but were not hazardous, uncomfortable, or embarrassing.
- Severe: fatal or life‐threatening, lowers the patient's life expectancy with severe impairment of a vital organ system; symptoms definitively hazardous to well‐being with significant impairment of function or incapacitation.

In addition, to provide a more helpful classification, a clinical significance was suggested for each adverse event. Table 1 shows the adverse events reported in the included studies and their classification.

**TABLE 1:** Suggested grouping classification and clinical relevance of the adverse events reported by the included studies.

Classification of adverse events reported in the included studies			
Clinical relevance	Classification	Adverse effects	
Not alter the patient's normal function	Trivial	Abnormal taste Abnormal thinking Anorexia Apathy Chills Constipation Discomfort Dry mouth	Euphoria Hypertonia Nervousness Perspiration Sore throat Sweating Thirst
	Mild	Anorexia Asthenia Blurred vision Confusion Drowsiness Dyspepsia Edema Heartburn Insomnia	Jitters Pain Pruritus Spots before eyes Tingling sensation Tinnitus Tiredness Vertigo
Some degree of impairment in function	Moderate	Allergic reaction Anemia Bleeding Chest pain Deep vein thrombophlebitis Diarrhea Dizziness Dysuria	Dyspnea Fever Gastrointestinal Headache Hypotension Nausea Paresthesia Skin reaction Sedation Vomiting
Lowers the patient's life expectancy/ life‐threatening	Severe	Death Liver and biliary system Renal failure Syncope	
Unknown	Other	Any other adverse effect not described previously or not reported in the study or reported as “other”	

All included studies used these four categories (Trivial, Mild, Moderate, and Severe) in the extraction data process. If the authors did not report any of these categories, the number of events in each category was 0. When the authors reported “other” adverse events, this category was compared separately because the severity of the events was unknown. Also, when the authors used multiple doses of the same drug in the comparator groups, the adverse events were summarized for all doses to facilitate a direct comparison with ketorolac.

### 2.7 Nomenclature of targets and ligands

Key protein targets and ligands in this article are hyperlinked to corresponding entries in http://www.guidetopharmacology.org, the common portal for data from the IUPHAR/BPS Guide to PHARMACOLOGY (Harding et al., 2018),^24^ and are permanently archived in the Concise Guide to PHARMACOLOGY 2019/20 (Alexander et al., 2019).^25^

## 3 RESULTS

### 3.1 Literature findings

**FIGURE 1:**
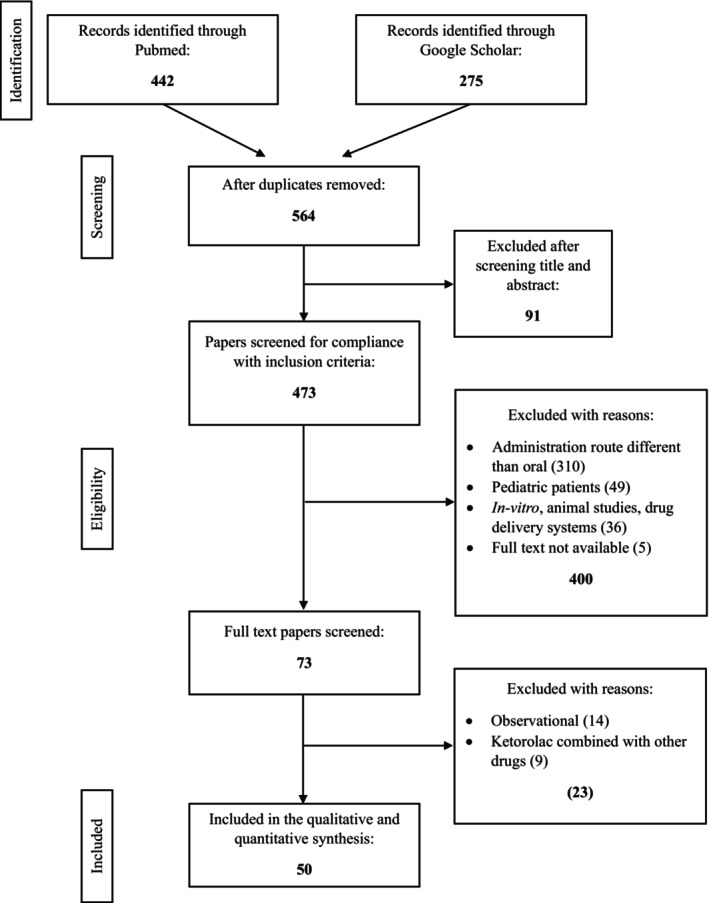
PRISMA flowchart of the systematic search process.

The systematic search process reveals approximately 717 potential studies in the databases. After removing duplicates, the total number of abstracts evaluated was 564. When the inclusion criteria were applied, 72 full‐text studies were analyzed. Fourteen observational studies and nine clinical trials in which ketorolac was used concomitantly with other drugs (see Table S1) were excluded. Finally, 50 studies^26^, ^27^, ^28^, ^29^, ^30^, ^31^, ^32^, ^33^, ^34^, ^35^, ^36^, ^37^, ^38^, ^39^, ^40^, ^41^, ^42^, ^43^, ^44^, ^45^, ^46^, ^47^, ^48^, ^49^, ^50^, ^51^, ^52^, ^53^, ^54^, ^55^, ^56^, ^57^, ^58^, ^59^, ^60^, ^61^, ^62^, ^63^, ^64^, ^65^, ^66^, ^67^, ^68^, ^69^, ^70^, ^71^, ^72^, ^73^, ^74^, ^75^ were included in the present systematic review and meta‐analysis (Figure 1); Table S2 summarizes each included study.

#### 3.1.1 Quality appraisal

As mentioned previously, the quality of the included studies was evaluated using the considerations reported by Pozos‐Guillén et al.^21^, ^22^ Table 2 shows the methodological characteristics of the included studies: 31 were randomized controlled trials, 6 were crossover, 4 were parallel, and 9 did not report the study design. The global quality value was 10.12, with a standard deviation (SD) of 1.93. The items “response variable” and “concordance of measuring method” were set as a value of 1 (qualitative objective and not clear, respectively) for all studies. Because in most of the included studies, the primary end point did not include the safety profile of ketorolac, the quality appraisal should be interpreted cautiously.

**TABLE 2:** Quality appraisal of the included studies.

Study	Trial design	Sample size calculation^a^	Randomization (homogeneous groups)^b^	Randomization method^c^	Blinding^d^	Follow‐up^e^	Response variable^f^	Concordance of measuring method^g^	Assumptions of statistical testing^h^	Results^i^	Total score
Bloomfield (1986)^26^	RCT	1	2	0	1	2	1	1	0	1	9
Honig (1986)^27^	NR	1	1	0	2	2	1	1	0	1	9
McQuay (1986)^28^	P	1	2	0	2	2	1	1	0	1	10
Johansson (1989)^29^	RCT	1	2	1	2	2	1	1	0	1	11
Carlson (1990)^30^	RCT + S	1	2	0	2	2	1	1	0	1	10
Smallman (1992)^31^	NR	1	2	0	0	2	1	1	0	1	8
Walton (1993)^32^	RCT	1	1	1	0	2	1	1	0	1	8
Wong (1993)^33^	RCT	2	2	1	2	2	1	1	0	1	12
Gebuhr (1994)^34^	P	1	1	0	0	2	1	1	0	1	7
Maslanka (1994)^35^	P	1	1	0	0	2	1	1	0	1	7
Naidu (1994)^36^	RCT	1	1	0	0	2	1	1	0	1	7
Nørholt (1995)^37^	NR	1	1	0	0	2	1	1	0	1	7
Ben‐David (1996)^38^	NR	1	1	0	1	2	1	1	0	1	8
Trombelli (1996)^39^	RCT	1	2	0	0	2	1	1	0	1	8
White (1997)^40^	RCT	2	2	1	2	2	1	1	0	1	12
Barber (1998)^41^	RCT	1	1	1	2	2	1	1	0	1	10
Innes (1998)^42^	RCT	2	2	2	2	2	1	1	1	1	14
Houry (1999)^43^	RCT	1	0	1	2	2	1	1	0	0	8
Pannuti (1999)^44^	CO	2	2	1	1	2	1	1	0	1	11
Sadeghein (1999)^45^	RCT	1	0	1	2	2	1	1	0	1	9
Olmedo (2001)^46^	RCT	1	2	0	2	2	1	1	0	1	10
Forrest (2002)^47^	RCT	2	2	1	0	2	1	1	0	1	10
Garibaldi (2002)^48^	NR	1	1	0	1	2	1	1	0	1	8
Compton (2003)^49^	CO	1	2	1	2	2	1	1	0	1	11
Rodriguez (2003)^50^	RCT	2	2	1	2	2	1	1	0	1	12
Costagliola (2008)^51^	CO	1	2	1	2	2	1	1	0	1	11
Aggarwal (2010)^52^	RCT	2	2	1	2	2	1	1	0	1	12
Isiordia‐Espinoza (2011)^53^	RCT	2	2	1	1	2	1	1	0	1	11
Mishra (2012)^54^	RCT	1	1	1	2	2	1	1	0	1	10
Sethi (2014)^55^	NR	2	2	1	0	2	1	1	0	1	10
Singh (2015)^56^	RCT	2	1	0	1	2	1	1	0	1	9
Yadav (2015)^57^	NR	1	1	0	2	2	1	1	0	1	9
Paiva‐Oliveira (2016)^58^	CO	1	2	1	2	2	1	1	0	1	11
Isiordia‐Espinoza (2016)^59^	RCT	2	2	1	1	2	1	1	0	1	11
Saha (2016)^60^	RCT	2	2	2	2	2	1	1	0	1	13
Crawford (2017)^61^	RCT	2	2	2	2	2	1	1	0	1	13
Meta (2017)^62^	RCT	1	1	1	0	2	1	1	0	1	8
Shah (2017)^63^	RCT	1	1	1	0	2	1	1	0	1	8
Kaladi (2019)^64^	RCT	2	2	1	0	2	1	1	0	1	10
Martins (2019)^65^	RCT	2	1	2	2	2	1	1	0	1	12
Serna‐Ojeda (2019)^66^	NR	1	1	1	0	2	1	1	0	1	8
Irizarry (2021)^67^	RCT	2	2	2	2	2	1	1	0	1	13
Kumar (2021)^68^	RCT	2	2	2	2	2	1	1	0	1	13
Martins‐de‐Barros (2021)^69^	CO	2	2	2	2	2	1	1	0	1	13
Sivasundaram (2021)^70^	P	2	2	1	0	2	1	1	1	1	11
Gupta (2022)^71^	NR	2	1	1	0	2	1	1	0	1	9
Mazhar (2022)^72^	CO	1	2	1	2	2	1	1	0	1	11
Rather (2022)^73^	RCT	1	2	1	0	2	1	1	1	1	10
Rao (2023)^74^	RCT	2	2	1	0	2	1	1	0	1	10
Elnaghy (2023)^75^	RCT	2	2	2	2	2	1	1	1	1	14

Abbreviations: CO, crossover design; NR, not reported; P, parallel design; RCT, randomized controlled trial, S, Sponsored.

^a^ 1, Unspecified/pilot study; 2, present.

^b^ 0, Not present; 1, not clear; 2, present.

^c^ 0, Unsuitable/not described; 1, adequate.

^d^ 0, Not described; 1, not clear/inappropriate; 2, present and described.

^e^ 0, Incomplete; 1, intention to treat/other analysis method; 2, full.

^f^ 0, Qualitative subjective; 1, qualitative objective; 2, quantitative.

^g^ 0, Not present; 1, not clear; 2, present/testing laboratory.

^h^ 0, Not present; 1, not clear/categorical data; 2, present and described.

^i^ 0, Incomplete; 1, complete.

### 3.2 Qualitative synthesis

Three studies^26^, ^27^, ^28^ evaluated ketorolac 5 mg versus active drugs (aspirin, diflunisal, and acetaminophen) and a placebo. The effect of the drugs was tested in patients in different clinical situations (postpartum, meniscectomy, and orthopedic), with a total of 90 patients exposed to oral ketorolac with a total of 5 trivial, 10 mild, 19 moderate, and 0 severe adverse events; two events were classified as other.

A total of 41 studies^26^, ^37^, ^40^, ^43^, ^44^, ^45^, ^46^, ^47^, ^48^, ^49^, ^50^, ^51^, ^52^, ^53^, ^55^, ^56^, ^57^, ^58^, ^59^, ^60^, ^62^, ^63^, ^65^, ^66^, ^67^, ^68^, ^70^, ^71^, ^72^, ^73^, ^74^, ^75^ reported the adverse events associated with the administration of ketorolac 10 mg versus active drugs (aspirin, diflunisal, acetaminophen, codeine, propoxyphene, diclofenac, ketobemidone, cetobemidone and a spasmolytic drug A29), morphine, dextropropoxyphene, ibuprofen, lornoxicam, hydrocodone, ketoprofen, dexketoprofen, tramadol, etodolac, dexamethasone, betamethasone, tapentadol, gabapentin, flupirtine, fentanyl, and meloxicam or a placebo in patients with various medical conditions (postpartum, meniscectomy, orthopedic, chemotherapy, third molar surgery, laparoscopic procedures, febrile illness, cancer pain, periodontitis treatment, major surgery, cold pressor test, glaucoma, dental pain, pulpitis, dental implant jaw prosthesis, corneal surgery, lower back pain, and dry socket). From all studies, a total of 7399 patients belonged to the ketorolac group and presented 27 trivial, 62 mild, 433 moderate, 18 severe adverse events, and 329 events classified as other.

The evaluation of 20 mg of ketorolac was carried out in eight studies^28^, ^39^, ^41^, ^46^, ^54^, ^61^, ^64^, ^69^ from those included in this review, making a comparison versus active drugs (acetaminophen, hydrocodone, ketoprofen, tramadol, ibuprofen, and dexamethasone) and a placebo in patients undergoing different conditions (orthopedic, periodontal treatment, third molar surgery, intrauterine device placement, and pulpitis). The studies reported a total of 274 patients receiving oral ketorolac; there were 6 cases of trivial, 5 mild, 83 moderate, 1 severe adverse events, and 12 classified as other. Only one study^38^ administrated a 30 mg dose of ketorolac versus placebo, in 14 patients with inguinal hernia and did not report any adverse event.

### 3.3 Quantitative synthesis

A risk ratio (RR) metanalysis was performed to quantitatively synthesize the adverse events reported in the included studies. Two subgroups were first defined to group the different studies: single or multiple intakes. Tablets of 10, 20, or 30 mg of ketorolac were defined as a single intake when only one intake of ketorolac was administered to the patients. Multiple intake of ketorolac was defined as more than one intake of ketorolac (Tablets of 10, 20, or 30 mg) administered to the patients at different times during the trial period. The second subgroup was the comparator, and the placebo group was defined as placebo administration in the comparator group. The active drug group was defined as the administration of any drug administered to the patients, including ketorolac, combined with any other drug simultaneously; the description of comparators in each included study is shown in Table S2.

### 3.4 Oral single intake of ketorolac versus placebo

**FIGURE 2:**
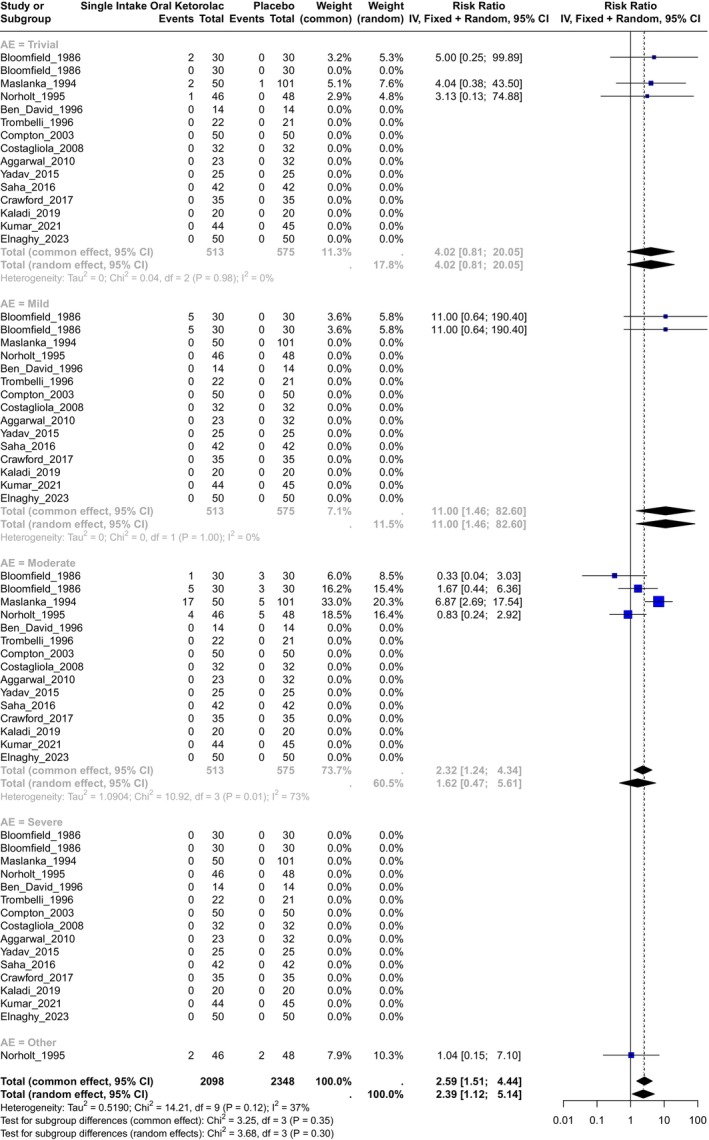
Forest plot of the comparison of single intake of oral ketorolac versus single intake of placebo.

A total of 14 studies evaluated this comparison^26^, ^35^, ^37^, ^38^, ^39^, ^49^, ^51^, ^52^, ^57^, ^60^, ^61^, ^64^, ^68^, ^75^ with ketorolac doses of 5, 10, 20, and 30 mg. Three of them^26^, ^35^, ^37^ were included in the calculation of the RR estimator, and 11 studies reported no adverse events in either groups (Figure 2). A total of 2098 observations were included in the ketorolac group and 2348 in the placebo group, with a total of 63 events. For the overall RR estimator, the inverse variance was calculated, and the heterogeneity was insignificant with *χ* ^2^ = 14.21, 9(d.f.) and *p* = 0.12 with I^2^ = 36.7%, and tau2 = 0.52. The common effect of RR for this comparison (including all adverse events) was **RR = 2.59**, **IC95% (1.51; 4.44)** with *p* = 0.02.

In the case of the **Trivial** adverse events, the common effect of **RR = 4.02**, IC95% (0.81; 20.05), and the heterogeneity was not significant with *χ* ^2^ = 0.04, 2(d.f.) and *p* = 0.98, I^2^ = 0%, and tau^2^ = 0. In the case of **Mild** adverse events, the common effect of **RR = 11**, IC95% (1.46; 82.6), and the heterogeneity was not significant with *χ* ^2^ = 0, 2(d.f.) and *p* = 0.1, I^2^ = 0%, and tau^2^ = 0. For **Moderate** adverse events, the random effect of **RR = 1.62**, IC95% (0.47; 5.61), and the heterogeneity was significant with *χ* ^2^ = 10.92, 3(d.f.) and *P* = 0.01, I^2^ = 73%, and tau^2^ = 1.09. For this comparison, the authors did not report a **Severe** adverse event, and only one study reported an **Other** effect. In summary, a single intake of oral ketorolac increased the risk of any adverse event significantly by 158.83% in a range of 51.02% to 343.6% compared with a placebo.

### 3.5 Oral multiple intakes of ketorolac versus placebo

**FIGURE 3:**
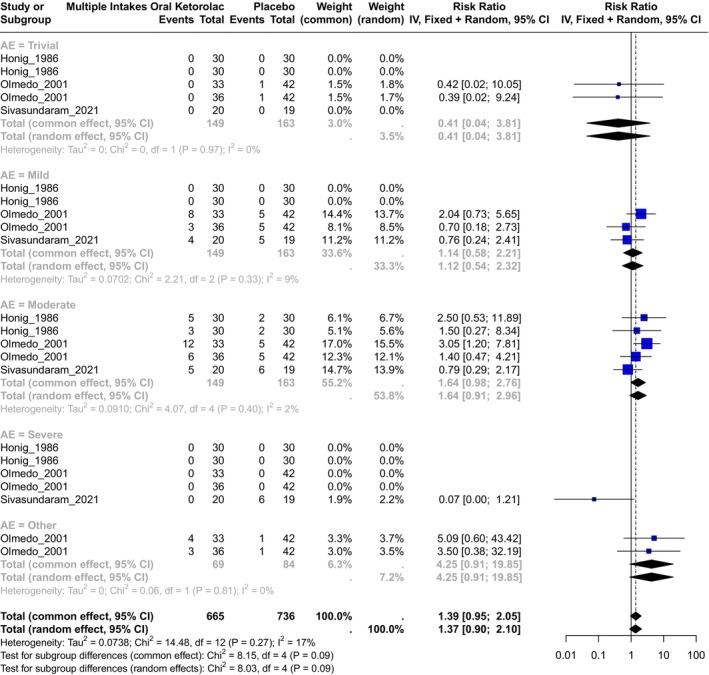
Forest plot of the comparison of multiple intakes of oral ketorolac versus multiple intakes of placebo.

A total of three studies evaluated ketorolac versus a placebo,^27^, ^46^, ^70^ with ketorolac doses of 5, 10, and 20 mg. All of them were included in the calculation of the RR estimator (Figure 3). A total of 665 observations were included in the ketorolac group and 736 in the placebo group, with 98 events. The schedule for ketorolac treatment was four intakes per day in 2, 3, or 5 days (see Table S2). The inverse variance was calculated for the overall RR estimator, and the heterogeneity was insignificant with *χ* ^2^ = 14.48, 12(d.f.) and *p* = 0.27 with I^2^ = 17.1%, and tau^2^ = 0.07. **The common effect of RR** for this comparison, including (all adverse events) was **RR = 1.39**, **IC95% (0.95; 2.05)** with *p* = 0.09.

In the case of the **Trivial** adverse events, the common effect of **RR = 0.40**, IC95% (0.04; 3.81), and the heterogeneity was not significant with *χ* ^2^ = 0, 1(d.f.) and *p* = 0.97, I^2^ = 0%, and tau^2^ = 0. For **Mild** adverse events, the common effect of **RR = 1.13**, IC95% (0.58; 2.21), and the heterogeneity was not significant with *χ* ^2^ = 2.21, 2(d.f.) and *p* = 0.33, I^2^ = 9%, and tau^2^ = 0.07. In the case of **Moderate** adverse events, the common effect of **RR = 1.64**, IC95% (0.98; 2.76), and the heterogeneity was not significant with *χ* ^2^ = 4.07, 4(d.f.) and *p* = 0.4, I^2^ = 2%, and tau^2^ = 0.09. Only one study reported **Severe** adverse events in the placebo group.^64^ In the case of adverse events classified as **Other**, the common effect of **RR = 4.25**, IC95% (0.91; 19.85), and the heterogeneity was not significant with *χ* ^2^ = 0.06, 1(d.f.) and *p* = 0.81, I^2^ = 0%, and tau^2^ = 0. In summary, multiple intakes of oral ketorolac increase the risk of any adverse event by 39.16% compared to a placebo; however, this effect is not significant.

### 3.6 Oral single intake of ketorolac versus active drugs

**FIGURE 4:**
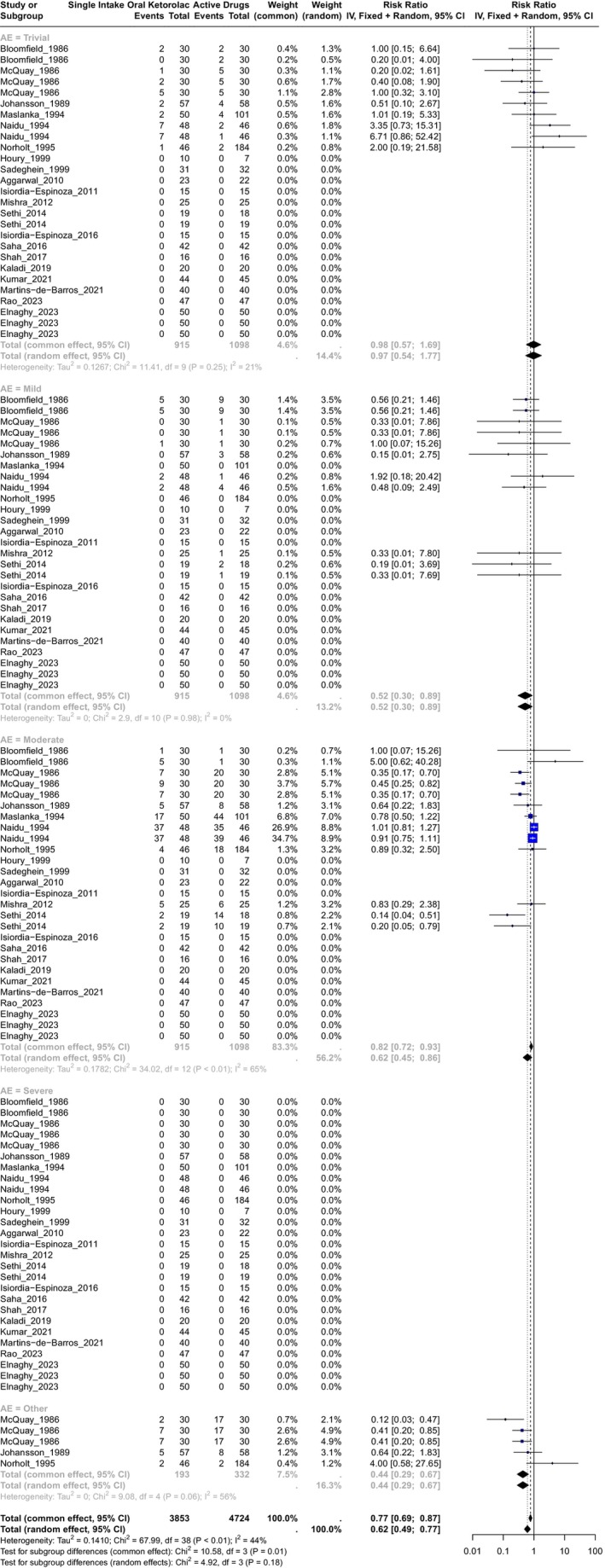
Forest plot of the comparison of single intake of oral ketorolac versus single intake of active drugs.

A total of 20 studies evaluated ketorolac versus active drugs,^26^, ^28^, ^29^, ^35^, ^36^, ^37^, ^43^, ^45^, ^52^, ^53^, ^54^, ^55^, ^59^, ^60^, ^63^, ^64^, ^68^, ^69^, ^74^, ^75^ with ketorolac doses of 5, 10, and 20 mg. Eight^26^, ^28^, ^29^, ^35^, ^36^, ^37^, ^54^, ^55^ were included in the calculation of the RR estimator, 12 studies reported no adverse events in either group (Figure 4). The active drugs used for this comparison were aspirin, acetaminophen (combined with dextropropoxyphene, ibuprofen, codeine, tramadol, ketorolac), morphine, lornoxicam, ibuprofen, ketorolac combined with tramadol, tramadol, etodolac, diclofenac, tapentadol, dexamethasone, and meloxicam. A total of 3853 observations were included in the ketorolac group and 4724 in the active drugs group, with a total of 567 events. For the overall RR estimator, the inverse variance was calculated, and the heterogeneity was significant with *χ* ^2^ = 67.99, 38(d.f.) and *p* = 0.002 with I^2^ = 44.1%, and tau^2^ = 0.141. **The random effects of RR** for this comparison (including all adverse events) was **RR = 0.62, IC95% (0.49; 0.77)** with *p* < 0.0001.

In the case of the **Trivial** adverse events, the common effect of **RR = 0.98**, IC95% (0.57; 1.69), and the heterogeneity was not significant with *χ* ^2^ = 11.41, 9(d.f.) and *p* = 0.25, I^2^ = 21%, and tau^2^ = 0.13. In the case of **Mild** adverse events, the common effect of **RR = 0.52**, IC95% (0.3; 0.89), and the heterogeneity was not significant with *χ* ^2^ = 2.9, 10(d.f.) and *p* = 0.98, I^2^ = 0%, and tau^2^ = 0. For **Moderate** adverse events, the random effect of **RR = 0.62**, IC95% (0.45; 0.86), and the heterogeneity was significant with *χ* ^2^ = 34.02, 12(d.f.) and *p* < 0.01, I^2^ = 65%, and tau^2^ = 0.18. For this comparison, the authors did not report a **Severe** adverse event. In the case of adverse events classified as **Other**, the random effects of **RR = 0.44**, IC95% (0.29; 0.67), and the heterogeneity was significant with *χ* ^2^ = 9.08, 4(d.f.) and *P* = 0.06, I^2^ = 56%, and tau^2^ = 0. In summary, a single intake of oral ketorolac reduces the risk of any adverse event significantly by 38.45% in a range of 50.99% to 22.7% when compared with active drugs.

### 3.7 Oral multiple intakes of ketorolac versus active drugs

**FIGURE 5:**
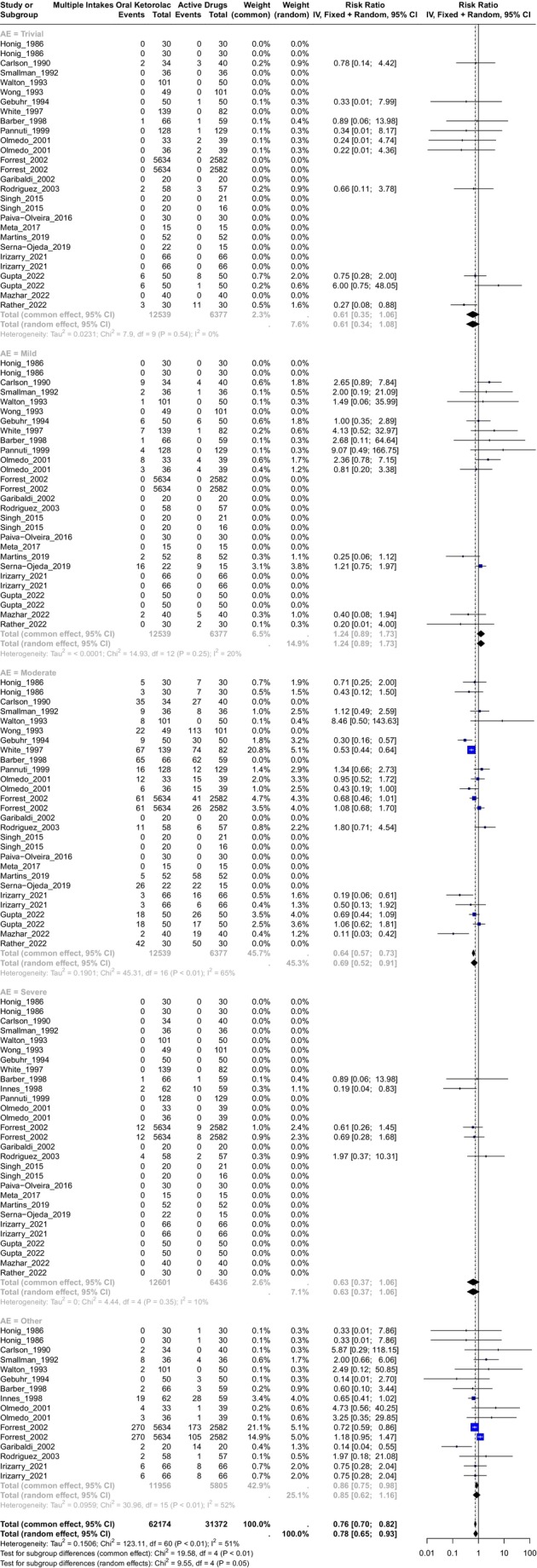
Forest plot of the comparison of multiple intakes of oral ketorolac versus multiple intakes of active drugs.

A total of 23 studies evaluated ketorolac versus active drugs,^27^, ^30^, ^31^, ^32^, ^33^, ^34^, ^40^, ^41^, ^42^, ^44^, ^46^, ^47^, ^48^, ^50^, ^56^, ^58^, ^62^, ^65^, ^66^, ^67^, ^71^, ^72^, ^73^ with ketorolac doses of 5, 10, and 20 mg. Nineteen^27^, ^30^, ^31^, ^32^, ^34^, ^40^, ^41^, ^42^, ^44^, ^46^, ^47^, ^48^, ^50^, ^65^, ^66^, ^67^, ^71^, ^72^, ^73^ were included in the calculation of the RR estimator (Figure 5). The active drugs used for this comparison were diflunisal, acetaminophen (combined with dextropropoxyphene, codeine, hydrocodone), diclofenac, ketobemidone (cetobemidone and a spasmolytic drug A29), ketoprofen, codeine, dexketoprofen, tramadol, dexamethasone, ketorolac combined with dexamethasone, gabapentin, ibuprofen, flupirtine, and transdermal fentanyl. A total of 62 174 observations were included in the ketorolac group and 31 372 in the placebo group, with 2330 events. The schedule for ketorolac treatment was 2, 3, or 4 daily intakes in a range period from 1 to 10 days (see Table S2). The inverse variance was calculated for the overall RR estimator, and the heterogeneity was insignificant with *χ* ^2^ = 123.11, 60(d.f.) and *p* < 0.0001 with I^2^ = 51.3%, and tau^2^ = 0.1506. **The random effects of RR** for this comparison (including all adverse events) was **RR = 0.78, IC95% (0.65; 0.93)** with *p* = 0.006.

In the case of the **Trivial** adverse events, the common effect of **RR = 0.61**, IC95% (0.35; 1.06), and the heterogeneity was not significant with *χ* ^2^ = 7.9, 9(d.f.) and *p* = 0.54, I^2^ = 0%, and tau^2^ = 0.231. For **Mild** adverse events, the common effect of **RR = 1.24**, IC95% (0.89; 1.73), and the heterogeneity was not significant with *χ* ^2^ = 14.93, 12(d.f.) and *p* = 0.25, I^2^ = 20%, and tau^2^ = <0.0001. In the case of **Moderate** adverse events, the random effects of **RR = 0.69**, IC95% (0.52; 0.91), and the heterogeneity was significant with *χ* ^2^ = 45.31, 16(d.f.) and *p* < 0.01, I^2^ = 65%, and tau^2^ = 0.1901. For **Severe** adverse events, the common effect of **RR = 0.63**, IC95% (0.37; 1.07) and the heterogeneity was not significant with *χ* ^2^ = 4.44, 4(d.f.) and *p* = 0.35, I^2^ = 10%, and tau^2^ = 0. In the case of adverse events classified as **Other**, the random effects of **RR = 0.85**, IC95% (0.62; 1.16), and the heterogeneity was significant with *χ* ^2^ = 30.96, 15(d.f.) and *p* < 0.01, I^2^ = 52%, and tau^2^ = 0.1. In summary, multiple intakes of oral ketorolac significantly reduce the risk of any adverse event by 21.99% in a range of 34.63% to 6.9% when compared with active drugs.

## 4 DISCUSSION

The present systematic review and meta‐analysis only included prospective clinical trials in adult patients using oral ketorolac. The grouping strategy covered two classifications: the comparator (active drug or placebo) and a single intake or multiple intakes. To simplify the grouping strategy, the single intake group included only the studies in which one dose (5, 10, 20, or 30 mg) of ketorolac was given to the patients. However, two studies^54^, ^63^ gave one dose of ketorolac before the third molar surgery and a second dose of ketorolac on the first day; these two studies were included in the single intake group. Additionally, Martins et al.^65^ used a single intake of a sublingual dose of ketorolac and gave patients one intake every 6 h for two postoperative days. This study was included in the multiple intake group. An appropriate one‐to‐one comparison was made to calculate the RR estimate from individual adverse events reported in each study.

An adverse effect related to drug therapy has been defined as “an appreciably harmful or unpleasant reaction resulting from an intervention related to the use of a medicinal product; an adverse effect usually predicts a hazard from future administration and warrants prevention, or specific treatment or alteration to dosage regimen, or withdrawal of the product.”^23^ For the present review, the term “adverse event” was used; adverse events can be classified into two types: Type A, a dose‐dependent augmented reaction related to the drug's pharmacodynamic profile, and Type B, an idiosyncratic and unpredictable reaction to the drug.^76^ Diagnosing adverse events is challenging for the clinician because these reactions can mimic the signs and symptoms of specific diseases and imply multiple characteristics such as temporality, dose‐related symptoms, intensity, and causal probability. To improve the applicability of the data, the adverse events were classified based on severity and clinical relevance (Table 1), and the effect size calculated was the risk ratio (RR). A risk ratio<1 implies risk reduction, and RR >1 implies an increased risk of any adverse event described in Table 1 and Table S2.

The most common adverse events related to the administration of ketorolac are summarized in a review article by Reinhart.^77^ These adverse events included hematological, gastrointestinal, renal, hypersensitivity, hepatic disease, and perinatal effects. The gastrointestinal adverse events related to ketorolac come from observational studies in which the endpoint was upper gastrointestinal (GI) bleeding. These cohorts were retrospective, and GI bleeding was associated with the previous consumption of NSAIDs by using patients without GI bleeding as the comparator group. These studies are reviewed by Castellsague et al., and the authors reported an RR >5 for GI bleeding associated with the administration of ketorolac.^10^ The main concern with observational studies is the uncertainty of the specific intake and doses of the drug reported by the patients and multiple confounders that complicate the true association between the drug and the adverse event.

The RR for postoperative bleeding has been previously analyzed by Gooble et al.^78^ In their review, the authors reported an RR = 1.1 IC95% (0.61; 2.06) and concluded that ketorolac did not increase the risk of postoperative bleeding and that the adverse events in the groups were not statistically significant with RR = 0.64 IC95% (0.41; 1.01).^78^ In our review, gastrointestinal and bleeding adverse events were classified as “moderate,” and the risk ratio for these adverse events was RR = 1.64, IC95% (0.98; 2.76) when oral ketorolac was compared with a placebo in multiple intakes, and RR = 0.69, IC95% (0.52; 0.91) when oral ketorolac was compared with active drugs in multiple intakes. It is important to note that the previous review reports were mostly made when ketorolac was administered parenterally. When oral ketorolac was compared with a placebo, the risk of moderate adverse events increased from 62% to 64%, but this risk increment was not significant. In contrast, when ketorolac was compared with active drugs, the risk of a moderate adverse event was reduced in a range of 38% to 31%.

For severe adverse events, Chang et al. in their review reported the risk–benefit profile of multiple NSAIDs, including ketorolac.^11^ Regarding renal function, the authors reported that using ketorolac for 5 days or less did not increase the rate of acute renal failure. However, if the administration of ketorolac continued for more than 5 days, the RR for renal failure was RR = 2.08 IC95% (1.08; 4).^79^ Gan et al. evaluated the cardiovascular safety of diclofenac by using a placebo and ketorolac as comparators, and reported a risk ratio for the cardiac event as RR = 1.04 IC95% (0.38; 2.90) when ketorolac was compared with a placebo and RR = 0.82 IC95% (0.38; 1.74) for vascular events when ketorolac was compared with a placebo.^80^ Also, note that, in this study, the ketorolac was administered intravenously.

In our study, severe adverse events were reported only when ketorolac was administered in multiple intakes. When ketorolac was administered in a single intake, the included studies did not report any severe adverse events. The risk ratio for severe adverse events was RR = 0.63, IC95% (0.37; 1.07) when ketorolac was administered in multiple intakes and compared with active drugs, meaning a risk reduction of 37.37%, while the RR was not significant. Remarkably, ketorolac tended to reduce the risk when compared with multiple active drugs; however, a direct comparison with specific drugs was not possible.

Although the present systematic review was focused on adult patients, the reviews of ketorolac safety in pediatric patients concluded that ketorolac is safe for postoperative pain management. The efficacy and safety of ketorolac for postoperative pain in pediatric patients was evaluated in a Cochrane review,^6^ However, the authors did not find sufficient data to analyze adverse events and serious events in this population. They also reported that the observed data related to the bleeding risk of ketorolac versus placebo were too low to conclude that ketorolac represents a higher risk for bleeding. Adverse events reported in the 13 studies included in this review were rare or very low.^6^ Marzuillo et al. in their review examine the pharmacokinetics, pharmacodynamics, efficacy, and safety of ketorolac in children and adolescents for the management of moderate to severe pain. The authors concluded that the main adverse events reported are at the gastrointestinal level when ketorolac is administered for a prolonged period and with preexistent risk.^13^

The safety of ketorolac has been chiefly associated with the dose and length of administration. Adverse events are most common, lasting over 5 days and occurring at higher doses.^4^ In the present systematic review, the maximum ketorolac administration time was 6,^73^ 7,^30^, ^33^, ^42^, ^44^, ^47^, ^50^ and 10 days,^34^ the RR for any adverse event of 10 mg of ketorolac versus active drugs with >6 days of treatment was RR = 0.8069 (0.6228; 1.0456), and the RR for any adverse event for 10 mg of ketorolac versus active drugs with >6 days of treatment was RR = 0.81 (0.62; 1.05).

By considering the characteristics of the patients, the correct choice of dose and administration time of oral ketorolac can result in effective pain management without significant side effects. The risk ratios found in the present systematic review and meta‐analysis were in the risk reduction direction (RR <1), except for trivial and mild adverse events (RR = 4 and RR = 11, respectively) when a single intake of oral ketorolac was compared with placebo.

The strengths and limitations of the present systematic review are as follows: this review includes only studies with the oral treatment of ketorolac, all studies are clinical trials, and some of them show methodological deficiencies; for example, 15 of the 50 included studies did not report the randomization method, 17 did not report the blinding process, and only few of them (four studies) shows the statistical assumptions for the data analysis; overall, methodological quality was 10.12, from a maximum score of 16 (see Table 2). The quality appraisal will be taken cautiously because, in most cases, the primary end point of the analgesic studies is not safety but generally its analgesic efficacy. Hence, the methodological design is not met to determine the safety profile of the analgesic drug. However, the advantage of using clinical trials instead of observational studies, even if the included studies had some methodological deficiencies, implies closer follow‐up to report adverse events, fewer confounders, and the posology of drug administration is more precise. Combining the different studies is always a challenge in systematic reviews because of the multiple schemes of administration, multiple doses of comparators, and different observation periods. However, the grouping method in our review allows direct comparisons to placebo or active drugs. Although the direct comparison with specific active drugs was not possible, it does not favor ketorolac because the comparisons include a wide variety of active drugs, implying a greater probability of adverse events in the ketorolac group than in each active drug in the comparator. This systematic review gives the clinical relevance of adverse events reported in the included studies (Table 1 and Table S2). This allows the clinician to interpret the RR reported here in a more helpful way for your daily practice.

## 5 IMPLICATIONS AND FUTURE DIRECTIONS

The evidence suggests that oral ketorolac is safe in pain scenarios when appropriately indicated. The present systematic review, group the adverse events related to the oral administration of ketorolac associated with their clinical relevance; however, the correlation between the event and the drug administration is still challenging for clinicians. Aronson and Ferner show the complexity of analyzing adverse drug events.^23^ Since the definition of adverse events, there are more complicated terms to use, like adverse effect, adverse reaction, or adverse drug event; each term has issues to clarify and depends on the patient experience and the clinician's interpretation. We proposed a helpful classification to define the adverse events related to ketorolac administration (described in Table 1); this classification can be used to report the adverse events related to drug administration more straightforwardly in conjunction with terminology unification; this facilitates data extraction and, consequently, direct drug comparisons. Finally, we encourage all authors to follow the CONSORT guidelines,^81^, ^82^ precisely item 19, and correctly report “harms” to facilitate the posterior data integration. We recommend a subsequent actualization of the present review and metanalysis with the incorporation of new clinical trials to up‐to‐date the risk ratios for the oral consumption of ketorolac and replicate the same process for the most common oral NSAIDs on the market.

## 6 CONCLUSION

Multiple intakes of 5, 10, and 20 mg of oral ketorolac, in treatment over 1–10 days, do not increase the risk of adverse events compared to placebo and show a tendency to reduce the risk of adverse events compared to active drugs. When a single intake of ketorolac (5, 10, 20, or 30 mg) is compared to a placebo, the risk increases only for trivial and mild adverse events.

## AUTHOR CONTRIBUTIONS

Esparza‐Villalpando Vicente: Conceptualization, Esparza‐Villalpando Vicente: Methodology, Esparza‐Villalpando Vicente and Ortiz‐Barroso Gladys and Masuoka‐Ito David: Data evaluation, Esparza‐Villalpando Vicente, Ortiz‐Barroso Gladys and Masuoka‐Ito David: Writing‐ Original draft preparation. Esparza‐Villalpando Vicente and Ortiz‐Barroso Gladys: Visualization, Investigation. Esparza‐Villalpando Vicente: Supervision. Esparza‐Villalpando Vicente: Validation. Esparza‐Villalpando Vicente, Ortiz‐Barroso Gladys and Masuoka‐Ito David: Writing ‐ Reviewing and Editing.

## CONFLICT OF INTEREST STATEMENT

Laboratorios Senosiain sponsored the present systematic review and meta‐analysis.

## ETHICS STATEMENT

Not applicable

## Supporting information


Table S1.

Table S2.

## Data Availability

The data that support the findings of this study are available from the corresponding author upon reasonable request.
