# Supplementary material for: Evidence‐based safety profile of oral ketorolac in adults: Systematic review and meta‐analysis
Source: Pharmacol Res Perspect. 2024 Nov 23;12(6):e70033. doi: 10.1002/prp2.70033 (PMC11584978; doi:10.1002/prp2.70033)
Supplement: Supplementary file 1 — Table S1. [file PRP2-12-e70033-s001.docx]

| Author (Year) | Reason of elimination | Conclusion |
| --- | --- | --- |
| Traversa (1995) | Observational study | Ketorolac had a higher risk of presenting gastrointestinal lesions compared to which other NSAIDs |
| García-Rodríguez (1998) | Observational study | The relative risk of upper gastrointestinal tract bleeding was higher in ketorolac than in other NSAIDs. The oral administration of ketorolac had a lower risk than the intramuscular administration, and a dose higher than 20 mg was used. |
| Menniti-Ippolito (1998) | Observational study | Ketorolac administration does not currently appear to be a public health problem in Italy, considering the hospitalizations for gastrointestinal issues related to the drug. |
| Pilotto (2003) | Observational study | Acute use in older people of ketorolac had a significant association with which gastrointestinal bleeding as, same as diclofenac, naproxen, nimesulide, and piroxicam. |
| Milo (2007) | Observational study | Patients after coronary artery bypass graft surgery had a significant improvement in survival when they also took aspirin. |
| Chang (2011) | Observational study | Parental use of ketorolac had a higher risk of lower gastrointestinal adverse events than oral ketorolac administration. |
| Castellsague (2013) | Observational study | Ketorolac has the highest relative risk compared to which other active drugs in upper gastrointestinal complications, especially in which a cumulative dose. |
| Kim (2015) | Observational study | Ketorolac had a higher risk of cardiovascular events compared to celecoxib, which had an increased risk of gastrointestinal bleeding. |
| Navarro-Martinez (2015) | Observational study | Ketorolac was not associated with agranulocytosis presence in a 10 mg dose. |
| Rafaniello (2016) | Observational study | Gastrointestinal events were more related to ketorolac exposure than other NSAIDs |
| Fjederhollt (2018) | Observational study | The occurrence of anastomotic leakage after surgery for gastro-esophageal-junction cancer was higher with ketorolac administration in within 7 days of administration. |
| McClain (2019) | Observational study | Oral ketorolac can be safely used in postoperative patients undergoing tonsillectomy and uvulopalatopharyngoplasty and does not increase postoperative hemorrhage rates. |
| Kachooei (2023) | Observational study | Oral 40 mg/day ketorolac administration can be safely used in the management of post-operative pain. |
| Forbes (1990) | Clinical Trial in which Ketorolac was combined with other drugs | No statistical differences were found in groups for side effects |
| Forbes (1990) | Clinical Trial in which Ketorolac was combined with other drugs | The number of patients reporting an adverse effect was higher for ketorolac and acetaminophen/codeine groups. |
| Toon (1990) | Clinical Trial in which Ketorolac was combined with other drugs | Interactions between ketorolac and warfarin are unlikely to be of significant clinical importance |
| Cold (1998) | Clinical Trial in which Ketorolac was combined with other drugs | Gravity and frequency of side effects were more significant during the ketorolac portion of the study; it is impossible to determine if this was as a result of lithium toxicity |
| Trindade (2012) | Clinical Trial in which Ketorolac was combined with other drugs | There  was no significant difference in the number of adverse side effects between groups. |
| Valle-Laisequilla (2012) | Clinical Trial in which Ketorolac was combined with other drugs | There were no adverse effects in the co-administration of ketorolac and hyoscine butylbromide for 48 hours. |
| Raja (2018) | Clinical Trial in which Ketorolac was combined with other drugs | Similar side effects occurred in both groups, but nausea and vomiting were higher in the control group. |
| Momesso (2021) | Clinical Trial in which Ketorolac was combined with other drugs | No adverse effects were reported |
| Mengers (2022) | Clinical Trial in which Ketorolac was combined with other drugs | The most common adverse effect reported was drowsiness, but no statistical differences existed between groups. |
