# Supplementary material for: Evidence‐based safety profile of oral ketorolac in adults: Systematic review and meta‐analysis
Source: Pharmacol Res Perspect. 2024 Nov 23;12(6):e70033. doi: 10.1002/prp2.70033 (PMC11584978; doi:10.1002/prp2.70033)
Supplement: Supplementary file 2 — Table S2. [file PRP2-12-e70033-s002.pdf]

Author (Year)	Study design	Characteristics of patients	Clinical condition	Concomitant treatment	Administration schedule	Study group description (n)	Oral dose	Tablets per day	Type of adverse event (n)	Observations
Bloomfield (1986) 26	Randomized double-blind trial	Female mean age 23 (3.5), ranged between 17-35 years	Healthy postpartum women	No use of analgesics in the previous 4 hours of the study.	Single oral doses	Ketorolac (30)	5 mg	Single dose	Drowsiness (3)<br>Dizziness (0)<br>Headache (0)<br>Nausea (1)<br>Sweating (1)<br>Euphoria (0)<br>Jitters (1)<br>Nervousness (1)<br>Spots before eyes (0)<br>Tingling sensation (0)<br>Tiredness (1)<br>Vomiting (0)	Side effects were reported by 30 patients, the most common was drowsiness, but there was no statistical difference between groups.
						Ketorolac (30)	10 mg	Single dose	Drowsiness (4)<br>Dizziness (2)<br>Headache (3)<br>Nausea (0)<br>Sweating (0)<br>Euphoria (0)<br>Jitters (0)<br>Nervousness (0)<br>Spots before eyes (0)<br>Tingling sensation (1)<br>Tiredness (0)<br>Vomiting (0)	
						Aspirin (30)	650 mg	Single dose	Drowsiness (8)<br>Dizziness (1)<br>Headache (0)<br>Nausea (0)<br>Sweating (1)<br>Euphoria (1)<br>Jitters (0)<br>Nervousness (0)<br>Spots before eyes (1)<br>Tingling sensation (0)<br>Tiredness (0)<br>Vomiting (0)	
						Lactose placebo (30)	-	Single dose	Drowsiness (4)<br>Dizziness (1)<br>Headache (0)<br>Nausea (1)<br>Sweating (0)<br>Euphoria (0)<br>Jitters (0)<br>Nervousness (0)<br>Spots before eyes (0)<br>Tingling sensation (0)<br>Tiredness (0)<br>Vomiting (1)	
Honig (1986) 27	Randomized double-blind clinical trial	Male (80.8%) and female (19.2%), mean age 32, ranged 18-50 years.	Patients who had undergone meniscectomy	Any analgesic drug was prohibited. Concomitant medication use	The ketorolac groups had 4 doses per day, diflunisal 2 times per day,	Ketorolac (30)	5 mg	4 tablets per day for 5 days	Gastrointestinal (1)<br>Headache (0)<br>Skin reaction (2)<br>Fever (1)<br>Other (0)	A total of 17 adverse effects were reported by 14 patients, most of them between days 2 and 5.

				was comparable between the groups	and placebo four times per day, for 5 days.	Ketorolac (30)	10 mg	4 tablets per day for 5 days	Gastrointestinal (3)<br>Headache (0)<br>Skin reaction (0)<br>Fever (0)<br>Other (0)	
						Diflunisal (30)	500 mg	2 tablets per day for 5 days	Gastrointestinal (4)<br>Headache (1)<br>Skin reaction (1)<br>Fever (1)<br>Other (1)	
						Placebo (30)	-	4 tablets per day for 5 days	Gastrointestinal (0)<br>Headache (2)<br>Skin reaction (0)<br>Fever (0)<br>Other (0)	
McQuay (1986)<br>28	Randomized clinical trial	Men and women between 20 and 70 years old	Patients undergoing elective orthopedic surgery.	All prescribed analgesic drugs were discontinued at least 3 hours before the study began.	Single dose	Ketorolac (30)	5 mg	Single dose	Headache (2)<br>Nausea (4)<br>Vomiting (0)<br>Sweating (1)<br>Thirst (0)<br>Blurred vision (0)<br>Dizziness (1)<br>Other (2)	The side effects were not severe enough that the patients discontinued the study, and there was no significant difference between any of the treatment groups.
						Ketorolac (30)	10 mg	Single dose	Headache (3)<br>Nausea (5)<br>Vomiting (0)<br>Sweating (1)<br>Thirst (1)<br>Blurred vision (0)<br>Dizziness (1)<br>Other (7)	
						Ketorolac (30)	20 mg	Single dose	Headache (2)<br>Nausea (3)<br>Vomiting (1)<br>Sweating (2)<br>Thirst (3)<br>Blurred vision (1)<br>Dizziness (1)<br>Other (7)	
						Acetaminophen (30)	100 mg	Single dose	Headache (5)<br>Nausea (6)<br>Vomiting (3)<br>Sweating (1)<br>Thirst (1)<br>Blurred vision (1)<br>Dizziness (0)<br>Other (8)	
						Acetaminophen (30)	500 mg	Single dose	Headache (3)<br>Nausea (2)<br>Vomiting (1)<br>Sweating (1)<br>Thirst (2)<br>Blurred vision (0)<br>Dizziness (0)<br>Other (9)	

Johansson (1989) 29	Randomized double-blind clinical trial	Patients' male (51%) and female (49%), mean age (SD) 41.2 (14.7) with good general health	Patients undergoing orthopedic surgery	Any drug before 6 hour the study was prohibited	Single dose and the adverse effects were registered in 6 hours	Ketorolac (57)	10 mg	Single dose	Dry mouth (1)<br>Headache (1)<br>Sweating (1)<br>Vertigo (0)<br>Nausea (2)<br>Gastro-intestinal pain (1)<br>Gastro-intestinal disorder (0)<br>Vomiting (0)<br>Sore throat (0)<br>Vasodilation (1)<br>Adverse events considered related to the medication (5)	One ketorolac-treated patient vomited, but the investigator considered it to be unrelated to the study medication.
						Doleron (58)	300 mg dextropropoxyphene napsylate, 700 mg aspirin and 300 mg phenazone	Single dose	Dry mouth (1)<br>Headache (0)<br>Sweating (1)<br>Vertigo (3)<br>Nausea (2)<br>Gastro-intestinal pain (0)<br>Gastro-intestinal disorder (1)<br>Vomiting (1)<br>Sore throat (2)<br>Vasodilation (0)<br>Adverse events considered related to the medication (8)	
Carlson (1990) 30	Randomized double-blind clinical trial	Male (53%) and female (47%) 18 years or older mean age 60.5 years (range 30-87).	Confirmed diagnosis of cancer and had moderate to severe intensity cancer pain for at least one week	The patients were allowed to receive chemotherapy or radiotherapy.	Administration was given 4 times daily for 7 days	Ketorolac (34)	10 mg	4 tablets per day for 7 days	Nausea (7)<br>Diarrhea (6)<br>Vomiting (3)<br>Gastrointestinal pain, dyspepsia (5)<br>Anorexia (1)<br>Constipation (1)<br>Headache (7)<br>Dizziness (4)<br>Somnolence (1)<br>Insomnia (2)<br>Dry mouth (0)<br>Pain (3)<br>Back pain (1)<br>Chills (1)<br>Facial edema (1)<br>Pruritus (0)<br>Rash (0)<br>Dyspnea (2)<br>Eye pain (1)<br>All others (2)	Adverse events were observed in both groups; nausea, vomiting and headache were the most common events in the ketorolac group.
						Acetaminophen/Codeine (40)	600 mg/60 mg	4 tablets per day for 7 days	Nausea (12)<br>Diarrhea (0)<br>Vomiting (5)<br>Gastrointestinal pain, dyspepsia (4)<br>Anorexia (1)<br>Constipation (1)<br>Headache (1)<br>Dizziness (2)	

									Somnolence (3)<br>Insomnia (0)<br>Dry mouth (2)<br>Pain (0)<br>Back pain (1)<br>Chills (0)<br>Facial edema (0)<br>Pruritus (1)<br>Rash (1)<br>Dyspnea (0)<br>Eye pain (0)<br>All others (0)	
Samallman (1992)<br>31	Randomized double-blind trial	Male (19.4%) and female (80.6%) over 65 years, mean age 75.1 (6.35)	Postoperative orthopedic hip, knee, ankle, or other surgery elderly patients	All patients receive a premedication 1 hour before surgery with papaveretum and atropine and general anesthesia with thiopentone and nitrous oxide. The study consists of an initial intramuscular phase with 10 mg papaveretum 3 hourly for 48 hours and Coproxamol for the oral phase for one group and Ketorolac 30 mg in the first dose and 10 mg for the other maximum of 15 doses in 48 hours and after that oral ketorolac.	In both groups, oral medication can be taken four times daily for 5 days.	Ketorolac (36)	10 mg	4 tablets per day for 5 days	Nausea and vomiting (3)<br>Indigestion (1)<br>Constipation (5)<br>Somnolence (0)<br>Confusion (2)<br>Other (8)	Nausea and vomiting were less frequent in the ketorolac group.
						Coproxamol (paracetamol plus dextropropoxyphene) (36)	Not reported	4 tablets per day for 5 days	Nausea and vomiting (1)<br>Indigestion (3)<br>Constipation (3)<br>Somnolence (1)<br>Confusion (1)<br>Other (4)	

Walton (1993) 32	Randomized double-blind clinical trial	Male (35.8%) and female (64.2%)	Surgical removal of impacted lower molars.	The test drugs were administrated through intramuscular injection in a single dose.	4 hours after the intramuscular dose, oral medication was given ketorolac 3 times a day (day 1) and 4 times a day (days 2 and 3), Diclofenac 2 times a day (day 1) and 4 times a day (days 2 and 3)	Ketorolac (101)	10 mg	3 or 4 times per day for 3 days	Nausea (3)<br>Dizziness (1)<br>Vomiting (3)<br>Dark urine (1)<br>Drowsiness (1)<br>Unsettled stomach (1)<br>Hot and cold flush (1)	Most of the adverse effects were probably not related to the medications.
						Diclofenac (50)	75 mg (day 1)<br>50 mg (days 2 and 3)	1 or 3 tiems per day for 3 days	Not reported	
Wong (1993) 33	Randomized double-blind multicenter clinical trial	Male (38.5%) female (61.5%)	Patients undergoing surgery with general anesthesia and be discharged from the hospital the same day.	The protocol consisted of an intravenous phase where the patients were located into 3 groups: Ketorolac (doses 1 and 2: 30 mg every 15 min, dose 3 to 8: 10 mg every 30 min), Fentanyl 50 (doses 1 and 2: 50 µg every 15 min, dose 3 to 8: 50 µg every 30 min), Fentanyl 10 (doses 1 and 2: 10 µg every 15 min, dose 3 to 8: 10 µg every 30 min)	For the oral phase, the patients in the ketorolac group continue with the same medication but orally every 4-6 hours for 7 days. Group Fentanyl 50 was given Codeine plus acetaminophen every 4-6 hours for 7 days as same as the Fentanyl 10 group.	Ketorolac (49)	10 mg	Tablets every 4 to 6 hours for 7 days	Nausea (4)<br>Vomiting (3)<br>Constipation (1)<br>Abdominal pain (0)<br>Headache (9)<br>Somnolence (3)<br>Dizziness (2)	Adverse effects probably related to drugs were more frequent in the groups of Codeine/Acetaminophen than ketorolac, but no statistically significant differences were observed.
						Codeine/Acetaminophen (55)	60 mg/600 mg	Tablets every 4 to 6 hours for 7 days	Nausea (21)<br>Vomiting (10)<br>Constipation (2)<br>Abdominal pain (3)<br>Headache (9)<br>Somnolence (8)<br>Dizziness (8)	
						Codeine/Acetaminophen (46)	60 mg/600 mg	Tablets every 4 to 6 hours for 7 days	Nausea (15)<br>Vomiting (6)<br>Constipation (3)<br>Abdominal pain (1)<br>Headache (7)<br>Somnolence (14)<br>Dizziness (6)	
Gebuhr (1994) 34	Randomized double-blind clinical trial	Male (59%) and female (41%), mean age 35.9 (1.8 SEM)	Patients post-operatively orthopedic surgery	Patients received an intramuscular injection of ketorolac or Ketogan every 1-6 hours as needed, as soon as clinically indicated, patients were	Maximum of 4 daily doses every 4-6 hours for 10 days	Ketorolac (50)	10 mg	4 tablets per day for 10 days	Insomnia (6)<br>Dizziness (1)<br>Headache (3)<br>Apathy (0)<br>Confusion (0)<br>Somnolence (0)<br>Nausea (3)<br>Vomiting (0)<br>Diarrhea (1)<br>Gastrointestinal pain (1)<br>Angina pectoris (0)	Significantly fewer patients in the ketorolac group experienced adverse effects in comparison with Ketogan. The most frequent was nausea.

				switched to oral doses					Chest pain (0)<br>Migraine (0)<br>Increased urinary frequency (0)<br>Urinary retention (0)<br>Asthenia (0)<br>Oedema (1)<br>Skin rash (0)	
						Ketogan (50)	5 mg of ketobemidone and 25 mg of N,;-dimethyl-3,3-diphenyl-1-methyl-allalamin chloride)	4 tablets per day for 10 days	Insomnia (5)<br>Dizziness (5)<br>Headache (3)<br>Apathy (1)<br>Confusion (1)<br>Somnolence (1)<br>Nausea (9)<br>Vomiting (7)<br>Diarrhea (1)<br>Gastrointestinal pain (0)<br>Angina pectoris (1)<br>Chest pain (1)<br>Migraine (1)<br>Increased urinary frequency (1)<br>Urinary retention (1)<br>Asthenia (1)<br>Oedema (0)<br>Skin rash (1)	
Maslanka (1994) 35	Randomized double-blind clinical trial	Male (71.6%) and female (28.4%), mean age 42.7 years (range 19-87)	Patients with moderate, severe, or very severe pain after hip or knee surgery	No other analgesic medication was allowed during the study and there was a 3-hour analgesic-free period before the study began.	A single dose of oral ketorolac or intramuscular morphine and evaluated for 6 hours.	Ketorolac (50)	10 mg	Single dose	Somnolence (12)<br>Dizziness (3)<br>Headache (1)<br>Hypertonia (1)<br>Sweating increased (1)<br>Dry mouth (0)<br>Fever (0)<br>Deep vein thrombophlebitis (1)<br>Nausea (0)<br>Dyspepsia (0)<br>Vomiting (0)	The incidence of adverse effects was similar in the drug groups. Ketorolac reported fewer adverse effects than morphine in an oral single dose.
						Morphine sulfate (51)	10 mg	Single dose	Somnolence (19)<br>Dizziness (5)<br>Headache (0)<br>Hypertonia (1)<br>Sweating increased (0)<br>Dry mouth (1)<br>Fever (1)<br>Deep vein thrombophlebitis (0)<br>Nausea (1)<br>Dyspepsia (0)<br>Vomiting (0)	
						Morphine sulfate (50)	5 mg	Single dose	Somnolence (13)<br>Dizziness (2)<br>Headache (0)<br>Hypertonia (0)<br>Sweating increased (0)<br>Dry mouth (0)<br>Fever (1)	

									Deep vein thrombophlebitis (0)<br>Nausea (0)<br>Dyspepsia (1)<br>Vomiting (0)	
						Placebo (25)	-	Single dose	Somnolence (2)<br>Dizziness (1)<br>Headache (1)<br>Hypertonia (0)<br>Sweating increased (1)<br>Dry mouth (0)<br>Fever (0)<br>Deep vein thrombophlebitis (0)<br>Nausea (0)<br>Dyspepsia (0)<br>Vomiting (1)	
Naidu (1994) 36	Randomized double-blind multicenter trial	Male (76.2%) and female (33.8%) mean age 35 (13)	Postoperative orthopedic or abdominal surgery patients with moderate to severe pain	Not reported	Single dose drugs, the subjects were observed for 6 hours.	Ketorolac (48)	10 mg	Single dose	Dizziness (13)<br>Dyspepsia (3)<br>Paresthesia (0)<br>Abnormal taste (3)<br>Somnolence (4)<br>Nausea (11)<br>Tinnitus (2)<br>Vomiting (6)<br>Sweating (3)<br>Abnormal thinking (1)	Dizziness, nausea, and vomiting were the most frequent in all groups, but none were serious or required treatment.
						Dextropropoxyphene/Paracetamol (46)	65 mg dextropropoxyphene plus 400 mg paracetamol	Single dose	Dizziness (4)<br>Dyspepsia (6)<br>Paresthesia (1)<br>Abnormal taste (0)<br>Somnolence (4)<br>Nausea (13)<br>Tinnitus (1)<br>Vomiting (7)<br>Sweating (1)<br>Abnormal thinking (1)	
						Ibuprofen/Paracetamol (49)	400 mg ibuprofen plus 325 mg paracetamol	Single dose	Dizziness (4)<br>Dyspepsia (10)<br>Paresthesia (0)<br>Abnormal taste (0)<br>Somnolence (0)<br>Nausea (17)<br>Tinnitus (4)<br>Vomiting (8)<br>Sweating (1)<br>Abnormal thinking (0)	
Nørholt (1995) 37		Male (57.3 %) and female (42.7 %), mean age 26.5 years range between 17-40 years	Patients after a mandibular third molar removal	Local anesthesia 2% Xylocain-adrenalin. Paracetamol was given as a rescue medication	Single dose, evaluation 6 hour after.	Ketorolac (46)	10 mg	Single dose	Dizziness (0)<br>Nausea, vomiting (1)<br>Abdominal pain or discomfort (1)<br>Headache (2)<br>Fever (0)<br>Discomfort (1)<br>Other (2)	The most common adverse events were dizziness, nausea, abdominal pain, headache, fever, and discomfort. No differences between groups.
						Lornoxicam (43)	4 mg	Single dose	Dizziness (1)<br>Nausea, vomiting (0)	

									Abdominal pain or discomfort (3)<br>Headache (1)<br>Fever (0)<br>Discomfort (0)<br>Other (0)	
						Lornoxicam (45)	8 mg	Single dose	Dizziness (2)<br>Nausea, vomiting (2)<br>Abdominal pain or discomfort (1)<br>Headache (0)<br>Fever (0)<br>Discomfort (0)<br>Other (1)	
						Lornoxicam (48)	16 mg	Single dose	Dizziness (3)<br>Nausea, vomiting (1)<br>Abdominal pain or discomfort (0)<br>Headache (0)<br>Fever (0)<br>Discomfort (1)<br>Other (1)	
						Lornoxicam (48)	32 mg	Single dose	Dizziness (1)<br>Nausea, vomiting (3)<br>Abdominal pain or discomfort (0)<br>Headache (1)<br>Fever (0)<br>Discomfort (1)<br>Other (0)	
						Placebo (48)	-	Single dose	Dizziness (1)<br>Nausea, vomiting (0)<br>Abdominal pain or discomfort (0)<br>Headache (1)<br>Fever (3)<br>Discomfort (0)<br>Other (2)	
Ben-David (1996)<br>38	Randomized double-blinded trial	Male (92.9%) and female (7.1%) mean age 49.32 years ASA I and II	Patients undergoing outpatient inguinal hernia repair	All patients received similar general anesthetic intraoperatively	Preoperative single dose	Ketorolac (oral) (14)	30 mg	Single dose	No side effects were reported	Ketorolac is a useful supplement to ilioinguinal plus field block regional anesthesia for hernia surgery.
						Ketorolac (intravenous) (14)	30 mg	Single dose		
						Ketorolac (intramuscular) (14)	30 mg	Single dose		
						Ketorolac (intrawound) (14)	30 mg	Single dose		
						Placebo (14)	30 mg	Single dose		
Trombelli (1996)<br>39	Randomized double-blind clinical trial	Males (30.2%) and females (69.7%) mean age 44 (8.87)	Patients with periodontal postoperative pain	All patient received local anesthesia 2% mepivacaine with 1:100000 epinephrine and Naproxen was allowed as a rescue medication	10 minutes prior the surgery patients receive a single dose of medication.	Ketorolac (22)	20 mg	Single dose	No side effects were reported	There were no adverse effects in the use of single 20-mg dose of ketrolac
						Placebo (21)	-	Single dose		

White (1997) 40	Randomized double-blind multicenter trial	Male (18.3%) and female (81.7%), mean age 33.33 (8)	Patients undergoing arthroscopic or laparoscopic tubal ligation procedures.	General anesthetic technique was induced with propofol (2-2.5 mg/kg) and fentanyl (100 µg) and maintained with isoflurane in combination 60% nitrous oxide in oxygen.	Single-dose phase 10 mg Multiple dose phase 10 mg every 4, 6 hours as needed for 3 days	Ketorolac (139)	10 mg	Tablets every 4 to 6 hours for 3 days	Nausea (26)<br>Vomiting (15)<br>Somnolence (17)<br>Dizziness (2)<br>Headache (6)<br>Pain (7)<br>Pruritus (1)	The only significantly higher adverse effect was postoperative dizziness in the patients receiving hydrocodone-acetaminophen.
						Hydrocodone and acetaminophen (82)	3.75 mg of hydrocodone and 375 mg of acetaminophen	Tablets every 4 to 6 hours for 3 days	Nausea (27)<br>Vomiting (15)<br>Somnolence (13)<br>Dizziness (11)<br>Headache (4)<br>Pain (1)<br>Pruritus (4)	
Barber (1998) 41	Randomized double-blind multicenter trial	Male (59.2%) and female (40.8%), age mean 30.5 (range 18 to 52 years)	Postoperative patients arthroscopically assisted, patellar tendon autograft reconstruction of the anterior cruciate ligament	Patients received at least one loading dose of parenteral ketorolac after the procedure. There was a 3-hour washout period between the last dose of an intermediate or long-term analgesic.	Medication was given 4 to 6 hours as needed for pain. The effects were evaluated 6 to 12 hours after dose 2.	Ketorolac (66)	Dose 1 consists of 20 mg, and dose 2 10 mg	Tablets every 4 to 6 hours for 12 hours	Chest pain (2)<br>Face edema (1)<br>Fever (3)<br>Headache (6)<br>Esophagitis (1)<br>Nausea (28)<br>Hypochromic anemia (1)<br>Dizziness (8)<br>Paresthesia (1)<br>Somnolence (5)<br>Pruritus (5)<br>Dysuria (1)<br>Infection (1)<br>Vomiting (4) Edema (1)<br>Nervousness (1)<br>Syncope (1)	54% of the patients in the ketorolac group experienced adverse events, 78% were classified as mild, 16% as moderate and only 5% as severe. Only 10% of the adverse events were probably related to ketorolac.
						Hydrocodone plus acetaminophen (59)	10 mg of hydrocodone and 1000 mg of acetaminophen	Tablets every 4 to 6 hours for 12 hours	Abdominal pain (1)<br>Headache (3)<br>Nausea (27)<br>Vomiting (6)<br>Dizziness (10)<br>Somnolence (7)<br>Hiccups (1)<br>Pruritus (4)<br>Sweating (1)<br>Amblyopia (1)<br>Cellulitis (1)<br>Fever (2)<br>Syncope (1)<br>Urinary retention (1)<br>Chest pain (1)<br>Dyspnea (1)	
Innes (1998) 42	Randomized clinical trial		Patients with moderate or severe musculoskeletal back pain of less than 72 hours duration	Patients in the ketorolac group requiring a fifth or sixth analgesic dose in 24 hours received	Administration was every 4 to 6 hours as needed in 24 hours	Ketorolac (62)	10 mg	Tablets every 4 to 6 hours for 7 days	Severe (2)<br>Other (19)	Adverse events included the digestive system and nervous system as vomiting, nausea, abdominal pain, dyspepsia, somnolence

				acetaminophen 650 mg in one or two doses.		Acetaminophen/Codeine (59)	600 mg/ 60 mg	Tablets every 4 to 6 hours for 7 days	Severe (10)<br>Other (28)	dysphoria, dizziness, and visual turbulence.
Houry (1999) 43	Pilot randomized double-blind study	Male (52.9 %) and female (47.1%), 18 years old and older.	Patients with febrile illness	Not receiving any other medication	Patients received single-dose and were evaluated at times 0, 30, 60, and 90 minutes	Ketorolac (10)	10 mg	Single dose	Allergic reaction to antibiotic	Only 1 patient with an allergic reaction; no side effects were reported in the first 90 minutes after administration of ketorolac.
						Acetaminophen (7)	750 mg	Single dose	Not reported	
Pannuti (1999) 44	Randomized double-blind cross-over trial	Male (47.4%) and female (52.6%) mean age 63 years ranged 30-71.	Cancer patients with moderate to severe pain	They did not receive concomitant chemotherapy and/or radiotherapy during the study or in the 10-15 days before the study	Patients receive the first medication 3 times daily for 7 days, then they cross over to the other treatment	Ketorolac (128)	10 mg	3 tablets per day for 7 days	Nausea (5)<br>Gastric pain (6)<br>Gastric pyrosis (3)<br>Diarrhea (0)<br>Pruritus (2)<br>Perspiration (0)<br>Colic pain (1)<br>Asthenia (2)<br>Vomiting (1)	Gastrointestinal effects were mainly observed when ketorolac was given as a second treatment after diclofenac.
						Diclofenac (129)	50 mg	3 tablets per day for 7 days	Nausea (3)<br>Gastric pain (5)<br>Gastric pyrosis (1)<br>Diarrhea (2)<br>Pruritus (0)<br>Perspiration (1)<br>Colic pain (0)<br>Asthenia (0)<br>Vomiting (1)	
Sadeghein (1999) 45	Randomized double-blind clinical trial	Male (59%) and female (41%) in age range of 16-60 years old	Acute periodontitis with severe pain	Patients who ingested analgesics within the last 4 hours of their visit were excluded.	Single dose and evaluated for 90 minutes after administration	Ketorolac (31)	10 mg	Single dose	No side effects were reported	There were no adverse effects in the use of ketorolac in the first 90 minutes.
						Acetaminophen/Codeine (32)	325 mg/15 mg	Single dose	No side effects were reported	
Olmedo (2001) 46	Randomized double-blind clinical trial	Male (34%) and female (64%), mean age 26.2 (9.85)	Patients after impacted third molar removal	Local anesthesia. Paracetamol was used as rescue medication	4 hours after surgery patients received medication every 6 hours during the first	Ketorolac (33)	10 mg	Tablets every 6 hours for 2 days	Drowsiness (6)<br>Pyrosis (5)<br>Dizziness (1)<br>Dyspepsia (3)<br>Bleeding (3)<br>Trismus (1)<br>Nausea (0)	The most common adverse effects reported were drowsiness, stomach disorders, dizziness, gastric heaviness and local bleeding

					2 postoperative days				Tremors (3)<br>Anorexia (2)<br>Sudation (0)<br>Vomiting (0)<br>Diarrhea (0)	
						Ketorolac (36)	20 mg	Tablets every 6 hours for 2 days	Drowsiness (3)<br>Pyrosis (2)<br>Dizziness (1)<br>Dyspepsia (1)<br>Bleeding (1)<br>Trismus (3)<br>Nausea (1)<br>Tremors (0)<br>Anorexia (0)<br>Sudation (0)<br>Vomiting (0)<br>Diarrhea (0)	
						Ketoprofen (39)	50 mg	Tablets every 6 hours for 2 days	Drowsiness (2)<br>Pyrosis (4)<br>Dizziness (3)<br>Dyspepsia (2)<br>Bleeding (2)<br>Trismus (0)<br>Nausea (2)<br>Tremors (1)<br>Anorexia (2)<br>Sudation (2)<br>Vomiting (1)<br>Diarrhea (1)	
						Placebo (42)	-	Tablets every 6 hours for 2 days	Drowsiness (5)<br>Pyrosis (1)<br>Dizziness (3)<br>Dyspepsia (0)<br>Bleeding (0)<br>Trismus (1)<br>Nausea (1)<br>Tremors (0)<br>Anorexia (0)<br>Sudation (1)<br>Vomiting (0)<br>Diarrhea (0)	
Forrest (2002) 47	Randomized multicenter trial	Male (39.5 %) and Female (60.5%) over 18 years old, age mean (SD) 49 (16.5).	Elective major surgery (orthopedic, abdominal, gynecological, urological, plastic ear, nose and throat, and others)	Ketorolac was parenteral 40-70 (IQR) mg/day for 2 days.<br>Patients can use an opioid if it is required.	Treatments were administrated according to the product label and varied by country.<br>All the drugs were administrated first parenteral maximum 2	Ketorolac (5634)	The maximum daily dose and duration of treatment were 40 mg/day for up to 7 days.	40 mg per day for 7 days	Death (9)<br>Surgical site bleed (61)<br>Gastrointestinal bleed (0)<br>Acute renal failure (3)<br>Allergic reaction (5)<br>Composite outcome (70)<br>Any outcome (77)<br>Other adverse reports (123)	Surgical site bleeding was the most frequent outcome, which was higher with the administration of postoperative anticoagulants.<br>However, there was no difference in adverse effects between ketorolac and the other drugs.

					days after the surgery, and then orally. Patients were followed for 30 days after their surgery.	Diclofenac (2582)	The maximum daily dose and duration of treatment were 150 mg/day for up to 7 days.	150 mg per day for 7 days	Death (5)<br>Surgical site bleed (37)<br>Gastrointestinal bleed (1)<br>Acute renal failure (4)<br>Allergic reaction (3)<br>Composite outcome (43)<br>Any outcome (48)<br>Other adverse reports (82)	
						Ketoprofen (3062)	The maximum daily dose and duration of treatment were 200 mg/day for up to 7 days.	200 mg per day for 7 days	Death (5)<br>Surgical site bleed (19)<br>Gastrointestinal bleed (3)<br>Acute renal failure (3)<br>Allergic reaction (4)<br>Composite outcome (26)<br>Any outcome (30)<br>Other adverse reports (49)	
Garibaldi (2002) 48	Randomized double-blind trial	Male (50.7%) and female (49.3%) aged between 18-32 years	Patients with bilateral third molar mandibular impactions	Sedation with a combination of 2.5 mg midazolam, with 25 mg meperidine and nitrous oxide anesthesia	Administration was maximally four times/day, for 5 days.	Ketorolac/placebo (20)	10 mg/0	4 tablets per day for 5 days	10% of patients with side effects	Of the patients taking ketorolac alone, only 10 % exhibited side effects. As the amount of codeine phosphate increased, the percentage of patient perceived side effects increased significantly
						Ketorolac/codeine (20)	10 mg/7.5 mg	4 tablets per day for 5 days	21% of patients with side effects	
						Ketorolac/codeine (20)	10 mg/15 mg	4 tablets per day for 5 days	25% of patients with side effects	
						Ketorolac/codeine (20)	10 mg/30 mg	4 tablets per day for 5 days	35% of patients with side effects	
						Placebo/codeine (20)	0/30 mg	4 tablets per day for 5 days	66% of patients with side effects	
Compton (2003) 49	Randomized double-blind crossover trial	Male (50%) and female (50%), mean age	Subjects on experimental cold pressor	Subjects didn't take any medications before the study	Patients received both treatments separated by at least 48 hours, and evaluated for 90 minutes after the administration	Ketorolac (50)	10 mg	Single dose	No side effects were reported	There were no adverse effects in a single 10 mg dose of ketorolac in a 90-minute period.
						Placebo (50)	-	Single dose		
Rodríguez (2003) 50	Randomized, double-blind clinical trial	Patients' male (76%) and female (24%) over 18 years, age mean (SD) 70 (9)	Diagnosis of malignancy and bone metastases, bone cancer pain lasting for more than 10 days.	Patients with another treatment within the last 15 days before the start of the study were excluded. Paracetamol (500 mg) plus codeine phosphate 30 mg were used	The patients take the medication every 6 hours for 7 (+1) days.	Ketorolac (58)	10 mg	Tablets every 6 hours for 7 (+1) days	Constipation (2)<br>Diarrhea (2)<br>Nausea/vomiting (4)<br>Gastrointestinal hemorrhage (1)<br>Other gastrointestinal adverse events (3)<br>Liver and biliary system (4)<br>Nervous system/psychiatric (0)<br>Skin and appendages (1)<br>Other (2)	The number of adverse effects was similar in both groups.

				as a rescue medication.		Dexketoprofen trometamol (57)	25 mg	Tablets every 6 hours for 7 (+1) days	Constipation (3)<br>Diarrhea (0)<br>Nausea/vomiting (2)<br>Gastrointestinal hemorrhage (0)<br>Other gastrointestinal adverse events (3)<br>Liver and biliary system (2)<br>Nervous system/psychiatric (1)<br>Skin and appendages (0)<br>Other (1)	
Costagliola (2008) 51	Randomized double-blind crossover trial	Male (53.12%) and female (46.88%), mean age 54.93 (6.98)	Patients affected by primary open-angle glaucoma and healthy volunteers.	Patients receiving topical ketorolac.	The patients receiving oral single dose of placebo and oral single dose of ketorolac, one week apart	Ketorolac (32)	10 mg	Single dose	No side effects were reported	There were no adverse effects in a single 10 mg dose of ketorolac.
						Placebo (32)	-	Single dose	No side effects were reported	
Aggarwal (2010) 52	Randomized double-blind trial	Male (52.2%) and female (47.3 %) mean age 31 years, (range 21-38)	Volunteers actively presenting pain in mandibular molar	Healthy and not taking any medication that altered pain perception. All patients received standard lidocaine and epinephrine injections	The patients take the medication one hour after the procedure	Ketorolac (23)	10 mg	Single dose	No side effects were reported	The study did not report side effects on patients.
						Ibuprofen (22)	300 mg	Single dose		
						Placebo (24)	Starch	Single dose		
Isiordia-Espinoza (2011) 53	Randomized double-blind clinical trial	Male (66.7%) and female (33.3%), 20.16 (1) mean age	Patients with diagnosis of impacted mandibular third molar	No other analgesics 24 hours before the procedure. Ketorolac swas given as a rescue analgesic.	Groups were given oral ketorolac plus intramuscular placebo or oral ketorolac plus intramuscular tramadol 30 min before the surgery, and evaluated for 24 h post-surgery	Ketorolac plus placebo (15)	10 mg	Single dose	No patients reported adverse events	There were no adverse events associated with the use of ketorolac as analgesic treatment for third molar surgery.
						Ketorolac/Tramadol (15)	10mg/50mg	Single dose		
Mishra (2012) 54	Randomized double-blind clinical trial	Male (48.64%) and female 51.36%) mean age 31.57	Patients undergoing third molar extraction	Local anesthetic 2% lignocaine was injected to all patients prior the extraction	The drugs were given 30 minutes before and after extraction	Ketorolac (25)	20 mg	2 tablets in 1 day	Sleepy (1)<br>Dizziness/ giddiness (2)<br>Weakness/tiredness (0)<br>Nausea/vomiting (2)<br>Tingling sensation (0)<br>Serios adverse events (0)	There were no significant differences between groups in adverse events

		years (range 18-65 years)				Tramadol (25)	100 mg	2 tablets in 1 day	Sleepy (1)<br>Dizziness/ giddiness (5)<br>Weakness/tiredness (1)<br>Nausea/vomiting (0)<br>Tingling sensation (0)<br>Serious adverse events (0)	
Sethi (2014) 55	Randomized double-blind clinical trial	Males (62.5%) and females (37.5%), mean age 30.72 years, aged between 18-60 years.	Symptomatic irreversible pulpitis	Patients most haven't taken medication in the last 8 hours. Local anesthesia with 2ml of xylocaine 2% with adrenaline 1:200,000	Single dose 30 minutes before endodontic treatment.	Ketorolac (19)	10 mg	Single dose	Nausea (0)<br>Vomiting (0)<br>Headache (1)<br>Dizziness (1)<br>Heartburn (0)	The ketorolac group reported fewer side effects than the other groups with a single dose of 10 mg.
						Tapendol (18)	100 mg	Single dose	Nausea (4)<br>Vomiting (2)<br>Headache (4)<br>Dizziness (4)<br>Heartburn (2)	
						Etodolac (19)	400 mg	Single dose	Nausea (1)<br>Vomiting (1)<br>Headache (4)<br>Dizziness (4)<br>Heartburn (1)	
Singh (2015) 56	Randomized double-blind clinical trial	Male (57.9%) and female (42.1%) over 18 years old, ASA I	Patients undergoing surgical removal of impacted mandibular molars	Local anesthesia. Amoxicillin was administrated 3 times daily to all groups, tab Rantac twice daily to groups with diclofenac and tramadol, and Tab Emeset twice daily to all groups.	Administration was 3 times per day for ketorolac and twice a day for Diclofenac and tramadol for five days post-operatively for	Ketorolac (20)	10 mg	3 tablets per day for 5 days	No adverse effects were reported	Authors did not report adverse effects.
						Diclofenac (21)	50 mg	2 tablets per day for 5 days		
						Tramadol (16)	50 mg	2 tablets per day for 5 days		
Yadav (2015) 57	Randomized double-blind controlled trial	Male (52%) and female (48%), age range 20.35 years	Patients with pain in a mandibular first or/and second molar	Patients were divided into two major groups alveolar nerve block with 1.8 ml 4% articaine with 1:100,000 epinephrine or 2% lidocaine with 1:80, 000 epinephrine, and then divided into 3 subgroups.	Ketorolac was given preoperatively.	Buccal infiltration/Lingua infiltration plus articaine or lidocaine (25)	0.9 ml each	Single dose	No adverse effects were reported	There were no adverse effects in this analgesic protocol.
						Ketorolac (25)	10 mg	Single dose		
						Ketorolac/ Buccal infiltration/Lingual infiltration plus articaine or lidocaine (25)	10 mg /0.9 ml each	Single dose		

Paiva-Oliveira (2015) 58	Randomized double-blind clinical trial	Healthy males (76.6%) and females (23.3%) individuals aged 20.8 years (1.80) ASA I	Patients undergoing extraction of the inferior third molars, one side receives ketorolac and the other side dexamethasone	Before surgery, patients received antisepsis treatment with 2% chlorhexidine topic and mouthwash with chlorhexidine 0.12%, and an anesthetic inferior alveolar, lingual, and buccal nerve blocks using articaine 4% with epinephrine.	Administration every 8 hours for two days	Ketorolac (30)	10 mg	Tablets every 8 hours for 2 days	No adverse effects were reported	There were no adverse effects in the use of ketorolac for 48 hours.
					Single initial dose, and then placebo every 8 hours for 2 days	Dexamethasone (30)	8 mg	Tablets every 8 hours for 2 days	No adverse effects were reported	
Isiordia-Espinoza (2016) 59	Pilot randomized double-blind clinical trial	Male (33.7%) and female (63.3%), mean age 22 (range 18-27)	Patients with impacted mandibular third molar.	No other analgesics 24 hours before the procedure. Ketorolac sublingual was given as a rescue analgesic	Groups were given oral ketorolac plus intramuscular placebo or oral placebo plus intramuscular tramadol 30 min before the surgery.	Ketorolac (15)	10 mg	Single dose	No patients reported adverse effects.	There were no adverse events reported in a single dose of ketorolac for third molar surgery.
						Tramadol (15)	50 mg	Single dose		
Saha (2016) 60	Randomized double-blind clinical trial	Male (51.5%) and female (48.5%), mean age 30.73 (7.83)	Patients with irreversible pulpitis	Inferior Alveolar Nerve Block (IANB) with 2% lidocaine containing 1:200,000 epinephrine	1 hour before administration of IANB	Ketorolac (42)	10 mg	Single dose	No adverse effects were reported	Adverse effects were encountered during the study with a single dose of ketorolac
						Diclofenac potassium (42)	50 mg	Single dose		
						Placebo (cellulose powder) (42)	-	Single dose		
Crawford (2017) 61	Randomized double-blind controlled trial	Female patient's mean age 31.3 (7.8)	Patients with pain after intrauterine device insertion	No other medications before insertion	Ketorolac was given 40-60 min before the procedure	Ketorolac (35)	20 mg	Single dose	No adverse effects were reported	There were no adverse effects in the single dose administration of ketorolac, before intrauterine device insertion.
						Placebo (35)	-	Single dose		

Meta (2017) 62	Randomized double-blind controlled trial	Males (36.7%) and females (63.3%), mean age 65.42, range between 43-81 years.	Subjects receiving a lower jaw hybrid prosthesis supported by 5 Osseo-Integrated root-form dental implants	Local anesthesia with chlorohydrate of carticaine 40 mgdL-adrenaline. Amoxicillin 3 times daily for 7 days	Ketorolac treatment twice daily for two days. Betamethasone 2 hours before surgery twice daily for 7 days	Ketorolac (15)	10 mg	2 tablets per day for 2 days	No adverse effects were reported	Authors did not report adverse effects in the study
						Ketorolac/Betamethasone (15)	10 mg/2 ml	3 tablets per day for 7 days		
Shah (2017) 63	Randomized clinical trial	Patients aged between 18-60 years.	Patients who required surgical extraction of impacted mandibular third molar	No other medication in 48 hours before the study.	Patients indicated to take the medication 1 hour after the procedure	Ketorolac (16)	10 mg	2 tablets per day	No adverse effects were reported	There were no adverse effects in the administration of 10 mg ketorolac.
						Tapentadol (16)	50 mg	2 tablets per day		
Kaladi (2019) 64	Randomized controlled trial	Male (61.7%) and female (38.3%), mean age 31.52 (4.06).	Irreversible pulpitis of any molar teeth, and good systematic health	Patients most haven't taken NSAID medication within 12 hours of the study. Lidocaine topical anesthesia was sprayed.	Patients received identical capsules 1 hour before administration.	Ketorolac (20)	20 mg	Single dose	No adverse effects were reported	There were no adverse effects in a single-dose of ketorolac.
						Ibuprofen (20)	400 mg	Single dose		
						Without premedication (20)	-	-		
Martins (2019) 65	Randomized triple-blind crossover clinical trial	Male (33%) and female (77%), mean age 20.8 (3.2), ASA I	Patients with bilateral third-molar removal	Local anesthesia mepivacaine 2% with epinephrine 1:100,000, acetaminophen and ondansetron were used as rescue medication	Administration was 1 hour before surgery and every 6 hours for 48 hours.	Ketorolac alone (52)	10 mg	Tablets every 6 hour for 2 days	Dizziness (0) Nausea (0) Vomit (0) Stomachache or other gastrointestinal discomforts (4) Migraine (1) Loss appetite (0) Drowsiness (2)	The total number of adverse events was higher in the concomitant treatment group and included dizziness, nausea, and vomiting
						Ketorolac/Tramadol/acetaminophen (52)	10 mg/37.5 mg/325 mg	Tablets every 6 hour for 2 days	Dizziness (11) Nausea (20) Vomit (13) Stomachache or other gastrointestinal discomforts (9) Migraine (5) Loss appetite (1) Drowsiness (7)	

Serna-Ojeda (2019) 66	Randomized clinical trial	Male (54%) and female (46%), mean age 24 (range 18-41) years	Patients undergoing epithelium-off or epithelium-on techniques	Topical anesthesia with tetracaine	The administration was every 8 hours for the first 3 post-operative days.	Ketorolac (22)	10 mg	Tablets every 8 hours for 3 days	Dizziness (9)<br>Drowsiness (14)<br>Headache (11)<br>Gastrointestinal symptoms (6)	No statistical difference was found between groups.
						Gabapentin (15)	300 mg	Tablets every 8 hours for 3 days	Dizziness (5)<br>Drowsiness (9)<br>Headache (11)<br>Gastrointestinal symptoms (6)	
Irizarry (2021) 67	Randomized double-blind trial	Male (62.7%) and female (37.3 %) patients mean age 39.73 (10.56)	Patients in the emergency department presenting moderate to severe lower back pain lasting more than 2 weeks	Not reported	Patients receiving medication every 8 hours as needed for 5 days	Ketorolac (66)	10 mg	Every 8 hours for 5 days	Any adverse effect (6)<br>Stomach irritation (3)	The side effects were similar in all groups and the most common was drowsiness. No serious side effects were attributable to investigational drugs.
						Ibuprofen (66)	600 mg	Every 8 hours for 5 days	Any adverse effect (8)<br>Stomach irritation (16)	
						Diclofenac (66)	50 mg	Every 8 hours for 5 days	Any adverse effect (8)<br>Stomach irritation (6)	
Kumar (2021) 68	Randomized double-blinded clinical trial	Males (55.2%) and females (44.8%), mean age 29 (8)	Patients with moderate to severe pain in the mandibular first or second molar	Anesthesia with g 1.8 ml of 2% lignocaine with 1:80,000 adrenaline	Preoperative single dose	Ketorolac (44)	10 mg	Single dose	No adverse effects were reported	Authors did not report adverse effects in the study
						Paracetamol (45)	650 mg	Single dose		
						Placebo (45)	-	Single dose		
Martins-de-Barros (2021) 69	Randomized triple-bind clinical trial	Males (32.5%) and females (67.5%), mean age 22.05 (2.47), ASA I	Patient with mandibular third molar removal	All participants received 1 g of amoxicillin orally and local anesthesia 2% mepivacaine with 1:100,000 adrenaline. Dipyrone monohydrate was used as a rescue drug.	Patients underwent to 2 surgical procedures, with washout intervals of at least three weeks dexamethasone was oral and ketorolac sublingual and was given 1 hour before the process.	Ketorolac (40)	20 mg	Single dose	No adverse effects were reported	There were no adverse effects in a single-dose ketorolac after 72 hours in mandibular third molar removal
						Dexamethasone (40)	8 mg	Single dose		
Sivasundaram (2021) 70	Randomized clinical trial	Male (66.7%) and female (33.3%) mean age 55.7 (10.6) years	Patients undergoing arthroscopic rotator cuff repair	Patients received an interscalene regional block with 30 cc of 0.5% bupivacaine with epinephrine, intravenpus dexamethasone	Ketorolac administration was every 6 hours for 3 days. Adverse effects was evaluated for 5 days	Ketorolac (20)	10 mg	Tablets every 6 hours for 3 days	Nausea/Vomiting (2)<br>Abdominal pain (1)<br>Diarrhea (0)<br>Constipation (0)<br>Indigestion, gastroesophageal reflux disease, gastritis (0)<br>Dark stools, rectal (0)<br>Allergy (1)<br>Dizziness/lightheadedness (0)	There was no statistical difference in adverse effects between groups.

				and ondansetron during surgery and analgesics in postacute care. Oxycodone-acetaminophen was prescribed every 4 tp 6 hours. Ketorolac group received intravenous ketorolac intraoperative and omeprazole once daily for 3 days post-operative					Drowsiness (1)<br>Insomnia (2)<br>Headache (2)<br>Dry mouth (0)<br>Itching (0)<br>Major adverse effect (0)	
						Control (19)	-	-	Nausea/Vomiting (2)<br>Abdominal pain (0)<br>Diarrhea (1)<br>Constipation (3)<br>Indigestion, gastroesophageal reflux disease, gastritis (0)<br>Dark stools, rectal (0)<br>Allergy (0)<br>Dizziness/lightheadedness (3)<br>Drowsiness (4)<br>Insomnia (1)<br>Headache (0)<br>Dry mouth (1)<br>Itching (1)<br>Major adverse effect (0)	
Gupta (2022) 71	Clinical trial	Male (62%) and Female (58%) mean age 38.75 (9.25) ranged 20-50 ASA I	Patients undergoing third molar removal	Local anesthesia	Patients have 3 doses of medication	Ketorolac (50)	10 mg	3 tablets per day for 5 days	Diarrhea (3)<br>Hypotension (2)<br>Nausea (9)<br>Sedation (0)<br>Sweating (6)<br>Sedation + hypotension (0)<br>Sweating + nausea (1)<br>Diarrhea + hypotension (3)	The side effects were higher in the tramadol group but not statistically different
						Tramadol (50)	50 mg	3 tablets per day for 5 days	Diarrhea (0)<br>Hypotension (7)<br>Nausea (4)<br>Sedation (11)<br>Sweating (8)<br>Sedation + hypotension (1)<br>Sweating + nausea (3)<br>Diarrhea + hypotension (0)	
						Flupirtine (50)	100 mg	3 tablets per day for 5 days	Diarrhea (1)<br>Hypotension (0)<br>Nausea (8)<br>Sedation (8)<br>Sweating (1)<br>Sedation + hypotension (0)<br>Sweating + nausea (0)<br>Diarrhea + hypotension (0)	
Mazhar (2022) 72	Randomized double-blind clinical trial	Male (60%) and female (40%), mean age 22.10 (3.15), ASA I	Patients with a surgical procedure for the removal of third molars	Local anesthesia 2% lignocaine with 1:80,000 adrenaline	Patients received oral ketorolac plus submucous	Ketorolac/Tramadol (40)	10 mg /50 mg	Tablets every 6 hours as needed for 1 day	Headache (5)<br>Nausea (8)<br>Itching (3)<br>Vomiting (3)<br>Pain at site injection (5)	The side effects were higher with the combination of ketorolac and tramadol.

					local tramadol or placebo	Ketorolac/Placebo (40)	10 mg	Tablets every 6 hours as needed for 1 day	Headache (1)<br>Nausea (1)<br>Itching (0)<br>Vomiting (0)<br>Pain at site injection (2)	
Rather (2022) 73	Randomized clinical trial	Males (43.3%) and females (56.7%), mean age 41.13 years ranged 18-60	Patients diagnosed with dry socket or post-extraction pain	Ketorolac patients received 20 mg first dose. Paracetamol was given as rescue medication	The patches were placed for 72 hours Ketorolac was given three times daily for 6 days.	Ketorolac (30)	10 mg	3 tablets per day for 6 days	Nausea (9)<br>Vomiting (3)<br>Somnolence (0)<br>Dizziness (3)<br>Headache (16)<br>Application site reaction (0)<br>Constipation (3)<br>Epigastric pain (11)	Vomiting and constipation were statistically higher in the fentanyl group, in contrast with epic gastric pain which was statistically higher in the ketorolac group.
						Transdermal fentanyl patch (30)	25 mcg/hr	A patch placement for 72 hours	Nausea (14)<br>Vomiting (13)<br>Somnolence (2)<br>Dizziness (9)<br>Headache (11)<br>Application site reaction (0)<br>Constipation (11)<br>Epigastric pain (3)	
Rao (2023) 74	Clinical trial	Males (43%) and females (57%), mean age 30.87 (4.40) years	The third molar impacted preoperatively patients	Rescue postoperative analgesic	Patients was given the drugs half an hour preoperatively.	Ketorolac (47)	10 mg	Single dose	No adverse effects were reported	There were no adverse effects in the use of preoperative ketorolac
						Tramadol (47)	50 mg	Single dose		
Elnaghy (2023) 75	Randomized double-blind controlled trial	Males (47.6%) and females (52.4%) mean age 32, 4 years.	Patients with moderate to severe pain diagnosed with symptomatic pulpitis of a mandibular first or second molar	All patient received injections and 0.9 mL long buccal injections containing 2% lidocaine and 1:100,000 epinephrine	Single oral premedication dose	Ketorolac (50)	10 mg	Single dose	No adverse effects were reported	The authors did not report the presence of adverse effect.
						Meloxicam (50)	7.5 mg	Single dose		
						Dexamethasone (50)	0.5 mg	Single dose		
						Ibuprofen (50)	600 mg	Single dose		
						Placebo (50)	-	Single dose		
